# Borg5 restricts contractility and motility in epithelial MDCK cells

**DOI:** 10.1242/jcs.261705

**Published:** 2024-12-10

**Authors:** David Cohen, Dawn Fernandez, Francisco Lázaro-Diéguez, Beatrix Überheide, Anne Müsch

**Affiliations:** ^1^ Albert-Einstein College of Medicine, Bronx, NY 10461, USA; ^2^ Proteomics Laboratory, Division of Advanced Research Technologies, New York University Grossman School of Medicine, New York, NY 10016, USA

**Keywords:** Borg5, Cdc42EP1, Epithelial cell morphology, Stress fiber types, Septin filaments, Myosin II

## Abstract

The Borg (or Cdc42EP) family consists of septin-binding proteins that are known to promote septin-dependent stress fibers and acto-myosin contractility. We show here that epithelial Borg5 (also known as Cdc42EP1) instead limits contractility, cell–cell adhesion tension and motility, as is required for the acquisition of columnar, isotropic cell morphology in mature MDCK monolayers. Borg5 depletion inhibited the development of the lateral F-actin cortex and stimulated microtubule-dependent leading-edge lamellae as well as radial stress fibers and, independently of the basal F-actin phenotype, caused anisotropy of apical surfaces within compacted monolayers. We determined that Borg5 limits colocalization of septin proteins with microtubules, and that like septin 2, Borg5 interacts with the rod-domain of myosin IIA (herein referring to the MYH9 heavy chain). The interaction of myosin IIA with Borg5 was reduced in the presence of septins. Because septins also mediate myosin activation, we propose that Borg5 limits contractility in MDCK cells in part by counteracting septin-associated myosin activity.

## Teaser

**Summary:** Borg (Cdc42EP) proteins are known to promote septin-dependent stress fibers and acto-myosin contractility. We show that MDCK Borg5 instead limits contractility and motility as required for acquisition of a mature epithelial phenotype.

## INTRODUCTION

Septins have emerged as versatile modulators of actin filament (F-actin) and microtubule (MT) dynamics (Lam and Calvo, 2019; Spiliotis, 2010, 2018; Spiliotis and Nakos, 2021). They exist in mammalian cells as palindromic heteromeric complexes composed of septin proteins from four sequence-homology groups (Field et al., 1996; Iv et al., 2021; Kinoshita, 2003; Kinoshita et al., 2002; Martins et al., 2023; Sellin et al., 2011, 2014; Sirajuddin et al., 2007); these building blocks can further anneal into filaments (Bridges et al., 2014; Field et al., 1996; Kinoshita et al., 2002; Woods and Gladfelter, 2021). The ability of septins to act as a modulator is thought to stem from three features: (1) septins can directly interact with F-actin and MTs (Dolat et al., 2014; Kuzmić et al., 2022; Mavrakis et al., 2014; Smith et al., 2015; Targa et al., 2019; Verdier-Pinard et al., 2017), which causes F-actin bundling and MT stabilization or growth, respectively (Bai et al., 2013; Bowen et al., 2011; Farrugia et al., 2020; Iv et al., 2021; Kuzmić et al., 2022; Nakos et al., 2022; Schmidt and Nichols, 2004); (2) septins bind membrane lipids and might provide a template for actin polymerization and possibly MT association with cellular membranes (Benoit et al., 2023; Dolat and Spiliotis, 2016; Hagiwara et al., 2011; Kinoshita et al., 1997; Martins et al., 2023); and (3) septins scaffold or compete with F-actin- and MT-modifying proteins resulting in a multitude of regulatory effects (reviewed in Spiliotis and Nakos, 2021). This diversity is enabled by the various septin paralogs interacting with modifying proteins. For example, septin 7 mediates recruitment of HDAC6 to MTs, which then become subjected to tubulin deacetylation (Ageta-Ishihara et al., 2013), whereas septin 2 competes with MAP4 for MT binding, which the authors linked, in HeLa cells, to increased MT-stability upon septin depletion (Kremer et al., 2005). Conversely, a splice variant of septin 9 (9_i1) functions as an MT-associated protein (MAP) and increases MT stability instead (Kuzmić et al., 2022). Given that septins 2 and 9_i1 coexist in the same septin complexes, their concomitant action could yield opposing effects on MT stability. In a similar apparent antagonism, septin 2 recruits myosin II and myosin-activating enzymes to F-actin (Joo et al., 2007), whereas septin 9 competes with myosin for actin binding (Smith et al., 2015). Such counteracting activities suggests that higher-order regulation defines the outcome, but such regulatory mechanisms have yet to be characterized.

Interaction of septins with the actin or MT cytoskeleton itself is regulated in vertebrates by Borg (for ‘binder of Rho GTPases’) proteins (Burbelo et al., 1999; Joberty et al., 1999, 2001), a family of five largely disordered proteins (Borg1–Borg5), which share a conserved α-helical domain that binds to the interface of septin 6 and 7 (Castro et al., 2023; Sheffield et al., 2003) as well as a Cdc42-binding Crib domain, which links Borg5-mediated septin regulation to Cdc42 activity (Farrugia and Calvo, 2017) and prompted the alternative denomination of Borgs as Cdc42 effector proteins (Cdc42EP1– Cdc42EP5). Available data suggest that Borg proteins enhance septin-mediated F-actin bundling, and promote septin-associated stress fibers and acto-myosin contractility (Calvo et al., 2015; Farrugia and Calvo, 2017; Farrugia et al., 2020; Hirsch et al., 2001). Borg2/3 depletion can even shift septin-association from F-actin to MTs (Salameh et al., 2021), supporting a view in which Borg proteins enable or facilitate F-actin associated septin functions (Farrugia and Calvo, 2016; Tomasso and Padrick, 2023). The molecular mechanisms underlying these regulations are still largely unknown. Only Borg2 has a putative actin-binding domain (Calvo et al., 2015), which could mediate enhanced septin–actin interaction; other possible mechanisms include Borg-induced changes in septin conformation, a scenario supported by observations of altered septin polymerization in the presence of Borg3 in cell-free assays (Soroor et al., 2021), and a hypothetical Borg-mediated recruitment of third-party proteins to septins.

Much of septin- and Borg-mediated F-actin regulation has been characterized in directionally migrating cells (Cohen et al., 2018; Farrugia and Calvo, 2017; Liu et al., 2014), in which the actin cytoskeleton underpins front–rear polarity which depends on three types of stress fibers: (1) transverse arcs intersecting with (2) radial stress fibers, which support the lamellae at the migratory front, and (3) ventral stress fibers providing contractility at the rear (Vallenius, 2013). Like directionally migrating cells, epithelial cells exhibit front-rear polarity when in an undifferentiated, mesenchymal state, that is during development, during mesenchymal transition in disease and during wound healing. Epithelial differentiation is associated with a dramatic switch from a motile or migratory phenotype to a stationary phenotype in which cells are isotropic, and largely lack stress fibers and protrusive lamella, but feature a circumferential actin belt linked to E-cadherin at zonula adherens (ZA) as well as actin filaments parallel to the lateral cortex (Lamouille et al., 2014; Morris and Machesky, 2015; Nelson, 2009; Scarpa et al., 2015; Thiery, 2002). The latter govern ‘columnarization’, an increase in cell height (Cavey et al., 2008; Kalaji et al., 2012). The role of septins in actin reorganization from a mesenchymal to epithelial pattern is not well understood. During epithelial differentiation, septins have been primarily linked to having a role in the MT-based machinery for the polarized delivery of exocytic membrane cargo, which is crucial for the establishment of distinct surface domain (Bowen et al., 2011; Spiliotis et al., 2008), and in supporting the actin association of cell–cell junctional complexes (Sidhaye et al., 2011; Wang et al., 2021). Likewise, despite evidence that Borg proteins are frequently mis-expressed in epithelial-derived cancers (reviewed in Tomasso and Padrick, 2023), and that Borg5 mutations are cancer drivers in oral squamous carcinoma (Campbell et al., 2021), Borg proteins have not yet been attributed any role in the establishment of the epithelial cytoskeletal networks. Indeed, unlike in other cultured cells, overexpression of Borg proteins in the kidney-derived epithelial model cell line MDCK fails to cause any obvious morphological changes or polarity defects (Hirsch et al., 2001). This prompted us to investigate how loss of function of endogenous Borg protein(s) in MDCK cells affects septin-dependent cytoskeleton organization and epithelial morphology. Given that available protein expression data suggested that MDCK cells express predominantly Borg5 (Cdc42EP1) (Harwood et al., 2023), which was also detected in a separate proximity ligation assay in the vicinity of apical junctional complexes in MDCKs (Tan et al., 2020), we focused our study on Borg5.

We found that Borg5 depletion prevented the acquisition of mature epithelial morphology and kept MDCK cells highly motile. In contrast to previously reported Borg functions, Borg5 in MDCK cells did not promote but instead limited stress fiber formation and acto-myosin contractility, and curbed MT-dependent lamella associated with mesenchymal migration. Identification of a large number of putative Borg5 interaction partners indicates that Borg5 has the ability to expand and/or modify the repertoire of septin-associated proteins.

## RESULTS

### Borg5 changes localization and expression levels during MDCK cell polarization

**Fig. 1.**
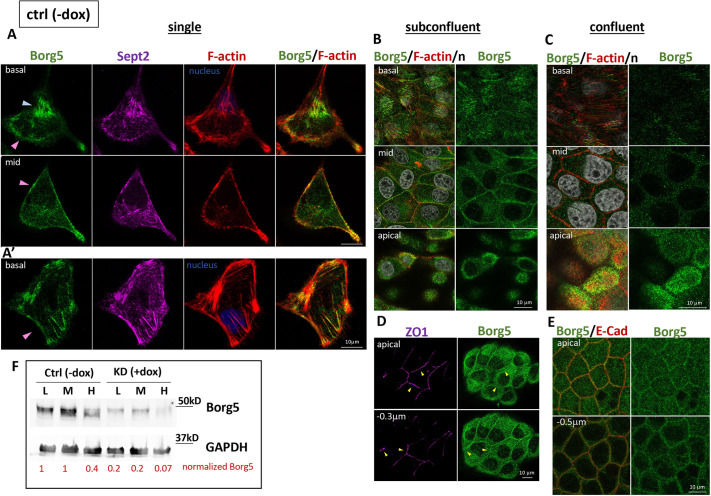
**Borg5 expression levels and localization change during MDCK polarization.** (A–E) MDCK Borg5 cells grown under control conditions without dox were plated at 2.5×10^3^ cells/cm^2^ for 3 h (A, single) on collagen-coated glass or at 10^5^ cells/cm^2^ (B,D, subconfluent) or 3×10^5^ cells/cm^2^ (C,E, confluent) for 20 h on uncoated glass (B–D) or for 48 h on Transwell inserts (E) and labeled for the indicated markers. Shown are confocal *x-y* sections at apical, sub-apical (−0.3 μm or –0.5µm), basal and mid-planes (mid). Arrowheads in A indicate subnuclear (blue) and cortical (pink) Borg5; arrowheads in D highlight non-overlapping localization of ZO1 and Borg5. (F) Borg5 IB of lysates from MDCK Borg5KD cells grown either without dox (ctrl, −dox) or induced with dox for Borg5 depletion (KD, +dox) and plated at the above low (denoted L), medium (denoted M) and high (denoted H) densities. Relative intensities, normalized with GAPDH, are indicated in red. All images are representative of three experimental repeats.

Immunofluorescence (IF) analysis (Fig. 1), the specificity of which we validated in Borg5-depleted MDCK cells stably expressing a dox-inducible Borg5 shRNAmir (immunoblot Fig. 1F; IF, compare −dox in Fig. 1A,C,E to +dox in Fig. S1A–C), revealed that Borg5 levels and distribution changed with density. In single cells, Borg5 localized in patches with septin 2 and stress fibers under the nucleus (Fig. 1A, basal, blue arrowhead) and where F-actin demarcated the lamellipodia-cell body interface (Fig. 1A, basal, pink arrowhead) or at F-actin bundles at the cell cortex (Fig. 1A′, pink arrowhead). Borg5 was also present at the free cortex above the attachment plane (Fig. 1A, mid). The Borg5 population under the nucleus decreased with confluency (Fig. 1B,C, compare basal plane images). In contacting subconfluent cells Borg5 also prominently localized along the lateral domain (Fig. 1B, mid, arrowhead) but was excluded from ZO1-labeled tight junctions (TJs; ZO1 is also known as TJP1) (Fig. 1D). With increasing polarization in mature monolayers, overall Borg5 levels decreased (Fig. 1F, compare −dox, M and H) and Borg5 accumulated at the apical domain (Fig. 1C, apical), and, when cells were grown on permeable filter substrates, at the ZA (Fig. 1E).

### Recombinant Borg5 recruits septins to the apical domain but does not increase septin-associated stress fibers

**Fig. 2.**
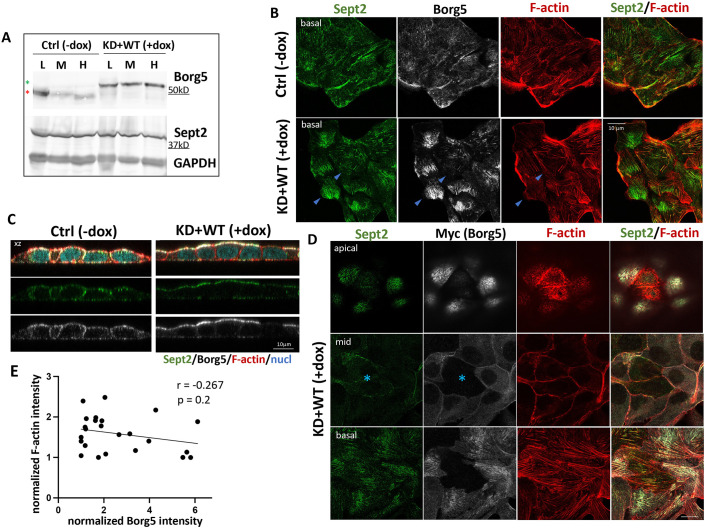
**Borg5 overexpression alters septin distribution but does not promote stress fibers.** Borg5KD+WT cells were grown either without dox (ctrl, −dox) or induced with dox for concomitant Borg5 depletion and expression of mouse Borg5–Myc (KD+WT, +dox) prior to plating at 2.5×10^3^ cells/cm^2^ (denoted L) 10^5^ cells/cm^2^ (denoted M) or 3×10^5^ cells/cm^2^ (denoted H). (A) Borg5 and Septin 2 IB with GAPDH for normalization. Red and green asterisks indicate the differential migration of endogenous (ctrl –dox) and recombinant (KD+WT +dox) Borg5. Note that recombinant Borg5 does not alter septin 2 levels. (B–D) Confocal *x-y* (B,D) or x-z (C) views of monolayers labeled for the indicated markers. Arrowheads in B point to cells with high and low basal Borg5 and Sept2 levels and their corresponding F-actin; the asterisks in D point to cells expressing low levels of Borg5 (note that these cells have more septin 2 in their mid-sections than surrounding cells expressing higher levels of Borg5). All images are representative of three experimental repeats. (E) Correlation analysis between ventral Borg5 and F-actin levels. A slope of −0.267 and *P*=0.2 suggest that stress fiber intensity is unrelated to Borg5 expression. Each data point from one cell with four cells from −dox and +dox in *N*=3, respectively; see Materials and Methods for analysis details.

To analyze the contribution of Borg5 to MDCK polarization, we generated knockdown (KD)-rescue pools of MDCK cells, in which the inducible shRNAmir was co-expressed with Myc-tagged RNAi-resistant mouse Borg5 (Borg5KD+WT). We established by immunoblotting, that the recombinant protein replaced endogenous Borg5 and that it was expressed above endogenous levels (Fig. 2A). IF analysis revealed that, even when overexpressed, Borg5–Myc localized like endogenous Borg5 to subnuclear stress fibers (Fig. 2B,D) and to lateral and apical domains, although the apical population was increased at expense of the lateral one (Fig. 2C, *z*-views). Overexpressed Borg5 caused recruitment of septin 2 to the apex (Fig. 2C, *z*-views). The higher Borg5 intensity under the nucleus also increased septin levels there (Fig. 2B), although this increase was not observed in mature monolayers [Fig. 2D, compare Sept 2 at the basal plane in cells expressing and not expressing (marked by asterisks) Borg5–Myc] and did not reflect an increase in total septin 2 levels (Fig. 2A, compare −dox and +dox). Importantly, increased Borg5 levels at the basal cortex did not translate into increased stress fiber formation (Fig. 2B,D, compare F-actin in cells with high and low Borg5 levels) and plotting Borg5 against stress fiber intensity at the basal cortex yielded no correlation between both (Fig. 2E). This suggests that recombinant Borg5 does not, as reported for other Borg proteins, promote septin-dependent stress fiber formation.

### Borg5 depletion in single cells yields cell morphology and F-actin organization similar to myosin II inhibition

The enrichment of Borg5 at several different subcellular domains hinted at multiple functions. To dissect them, we first investigated Borg5KD phenotypes in single cells 3 h after seeding on collagen-coated coverslips.

**Fig. 3.**
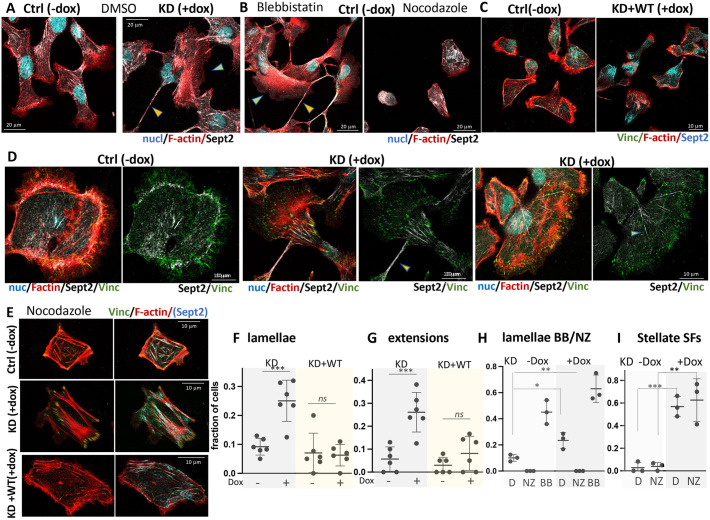
**Borg5 KD in spreading cells shifts the balance between MT- and MyoII-dependent processes.** (A–E) Induced Borg5 KD cells (KD, +dox) (A,B,C,E) or Borg5KD+WT cells (KD+WT, +dox) (C,E) and their corresponding uninduced controls (ctrl, −dox) were plated as single cells as described for Fig. 1A either without manipulations (C,D) or in the presence of DMSO solvent, 10 µM nocodazole (NZ) (A,B,E) or 20 µM blebbistatin (BB) (A,B) and labeled as indicated. Presented are confocal *x-y* sections. Yellow and cyan arrowheads in A and B point to lamellae and cell extensions, respectively. (F,G) Fraction of Borg5KD and KD+WT cells in −dox or +dox conditions with lamellae (F) or extensions (G). (H,I) fraction of Borg5KD cells in −dox or +dox conditions with lamellae upon NZ or BB treatment (H) and with stellate stress fibers (I). Stellate stress fibers (SFs) were identified as F-actin bundles unrelated to peripheral circumferential F-actin as in the KD+dox panel in E. (F–I) *N*=3, 20 cells from random image fields of 40× objective images. ***P=0.0005 (F); 0.0006 (G), 0.0008 (I); **P=0.003 (H), 0.006 (I); *P=0.024 (H); n.s., not significant (unpaired two-tailed *t*-test). Error bars show mean±s.d.

MDCK morphology upon attachment results, as in other cell types (Even-Ram et al., 2007; Sato et al., 2020), from the outcome of two antagonistic processes: (1) Rho-dependent acto-myosin II-activity, which promotes circumferential actin bundles and restricts spreading; it dominates when MTs are disrupted by nocodazole treatment [Fig. 3B, Ctrl (−dox), nocodazole], and (2) MTs targeting the cortex and activating Rac1, which causes formation of large lamellae as well as MT-dependent neurite-like extensions; it dominates when myosin II is inhibited by blebbistatin [Fig. 3B, Ctrl (−dox), blebbistatin] (Even-Ram et al., 2007; Rafiq et al., 2019; Sato et al., 2020). In untreated Borg5KD −dox controls, most cells featured small lamellae and there were few cell extensions (Fig. 3A, −dox, DMSO; quantification in Fig. 3F,G, KD −dox). Borg5-depleted cells, by contrast, were more likely to form large lamellae and extensions like blebbistatin-treated cells (Fig. 3A, +dox DMSO; quantification in Fig. 3F,G, KD +dox), suggesting that MT-activities prevailed. Because septins can support either F-actin or MT organization, we hypothesized that Borg5 depletion altered the allocation of septins to the two cytoskeletal systems. Indeed, whereas septin 2 was loosely aligned with cortical and internal F-actin in control cells (Fig. 3D, −dox, Fig. 1A,A′; Fig. S2C), septin populations distinct from F-actin organization were apparent in Borg5KD cells (Fig. 3D, +dox; Fig. S2D,E); they either radially emanated from the cell center (Fig. 3D, KD+dox; Fig. S2D, blue arrowheads) or concentrated in neurite-like cell extensions (Fig. 3D, KD+dox, yellow arrowhead). We quantified this phenomenon in Fig. S2D′ by scoring the septin organization in cells as being either aligned with or distinct from the trajectories of main F-actin bundles. A quantitative pixel-by-pixel colocalization analysis proved less meaningful in this case, because septins only approximate rather than precisely colocalize with a subset of F-actin fibers (Martins et al., 2023). Colabeling for septin 2 with tubulin and F-actin, or of septin 9 with acetylated tubulin and F-actin, furthermore indicated that in Borg5KD cells, septin filaments aligned with MTs rather than F-actin when both cytoskeletal systems were present in the same plane (Fig. S2E,D, blue arrowheads), whereas in control cells septins appeared largely distinct from MT trajectories (Fig. S2B,C).

To determine whether Borg5-dependent differences in MT–septin alignment account for the observed differences in morphology and F-actin organization, we compared control and Borg5KD cells under conditions of nocodazole-mediated MT disruption. Nocodazole treatment during spreading resulted in robust septin alignment with F-actin in both control and Borg5KD cells (Fig. 3E, ctrl −dox, KD+dox), supporting the notion that MTs compete with F-actin for septin binding. Borg5-depleted cells nevertheless failed to organize the cortical F-actin bundles characteristic of control cells; they instead frequently accumulated parallel or stellate stress fibers in the cell center, linked to robust vinculin-positive focal adhesions (Fig. 3E, KD+dox; quantified in Fig. 3H,I). This indicates that Borg5-mediated suppression of MT activities alone does not account for the Borg5KD phenotype and suggests that Borg5 has additional MT-independent roles in F-actin organization.

Co-expression of the Borg5 shRNAmir with WT Borg5 prevented increased lamella formation and outgrowth of neurite-like extensions (Fig. 3C,F,G, KD+WT), the aberrant stellate stress fibers after nocodazole treatment (Fig. 3E, KD+WT) and increased septin–MT alignment (Fig. S2A,A′), indicating that the phenotypes were indeed due to Borg5 depletion and furthermore revealing that MDCK morphology in single cells is not altered by elevated Borg5 levels.

Taken together, in single cells, Borg5 restricts MT-dependent lamellae and promotes the establishment of cortical actin bundles as opposed to a cell center-based actin network.

### Borg5 depletion causes contractile MDCK monolayers

**Fig. 4.**
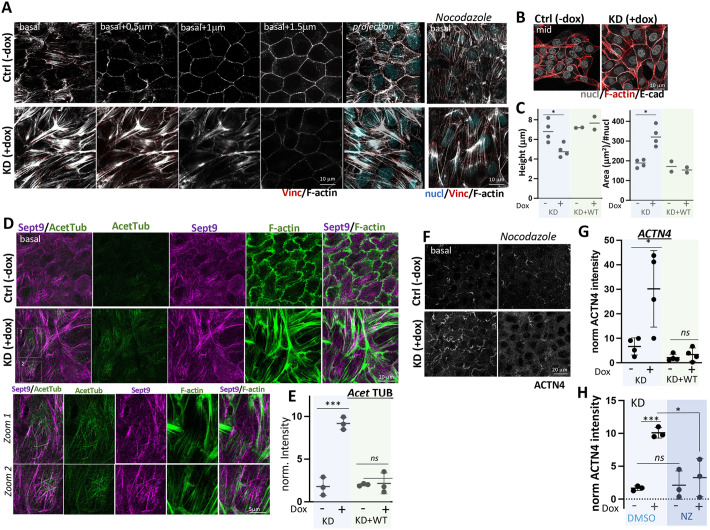
**Borg5 depletion in developing monolayers promotes radial stress fibers and stabilized MTs and impairs lateral membrane development.** (A,B,D,F) Uninduced or dox-induced Borg5KD plated at either 3×10^5^ (A,D–E) or at 10^5^ cells/cm^2^ (B,C) were cultured for 24 h and, where indicated, treated for 2 h with 10 µM nocodazole (NZ) or DMSO solvent. Confocal sections of IF labeled Borg5KD cells in −dox or +dox conditions at the basal domain (basal, A,D,F), sections +0.5, +1 and 1.5 µm above the basal domain (A, basal +0.5 to 1.5), mid-sections across the nucleus (mid, B) or projections excluding the apical surface (A, projection). Note in A, the paucity of basal circumferential cortical F-actin in Borg5KD cells (arrowheads) and the location of stellate stress fibers under the nucleus, apparent in NZ-treated cells. Note in D, that stabilized (acetylated) tubulin at the basal domain in KD+dox, but not in ctrl cells, aligns with segments of septin 9 whereas septin 9 and F-actin have distinct patterns (zoomed images of boxed regions). Note in F, the differences in peripheral ruffles. (C) Compaction in isolated cell islands determined by area/nuclei (normalized area), and by cell height in induced and uninduced Borg5KD and KD+WT cells. The area of five random islands per sample was determined at the *z*-plane of the nuclear center (as shown in B) and divided by the number of nuclei; height was determined by the number of top-to-bottom 0.3 µm *z*-sections; *N*=3 for KD, *N*=2 for KD+WT. *P=0.029 (height), *P*=0.02 (area) (Mann–Whitney *t*-test). See Fig. S5 for IF images of the quantified KD+WT samples. (E,G,H) Integrated fluorescence intensities of basal acetylated tubulin (E) or ACTN4 (G,H) in Borg5KD and KD+WT cells (E,G) or in Borg5KD cells treated with DMSO or NZ (H) measured from three random images and normalized to the lowest number in each dataset; *N*=3 (E,H), *N*=4 (G). ***P=0.0006 (E), 0.0001 (H); *P=0.026 (G), 0.017 (H); n.s., not significant [*P*=0.8 (E), *P*=0.44 (G), *P*=0.74 (H)] (unpaired two-tailed *t*-test). See Fig. S5 for IF images for the quantified KD+WT samples. Error bars show mean±s.d.

Aberrant stellate stress fibers also characterized Borg5-depleted cells when they were plated at confluency (Fig. 4A). Like the situation in single cells, MT disruption did not abolish the stress fiber phenotype (Fig. 4A, nocodazole); the heightened cell isotropy in nocodazole-treated cells (see Fig. 6B,C) clarified, however, that the stellate stress fiber hub localized to the cell center under the nucleus, with the emanating stress fibers ending in focal adhesions, which aligned between neighboring cells at the periphery. Active myosin II, as measured with an antibody to phosphorylated myosin light chain (ppMLC; herein referring to MLC2 phosphorylated at Thr18 and Ser19) was enriched at the stress fiber hub (Fig. S3A,B), and overall ppMLC intensity at the basal domain was greatly increased compared to that in controls (Fig. S3C,D). Acto-myosin asters resembling this stellate organization can form *in vitro* through myosin II-mediated F-actin polarity sorting whereby unidirectional bipolar myosin filaments gather F-actin filaments at their plus ends (Köster et al., 2016; Miller et al., 2018). In MDCK (Nakano et al., 1999) and HeLa cells (Ishizaki et al., 1997), stellate stress fiber organization with strong FAs, as seen in Borg5KD cells, was triggered by expression of an activated form of Rho kinase 1 (ROCK1). We found that Rho kinase (ROCK; herein referring to both ROCK1 and ROCK2) but not myosin light chain kinase (MLCK) inhibitors abolished the stress fibers in Borg5KD cells (Fig. S3C,D). Therefore, Borg5 depletion likely exaggerates cortical stress fiber formation under the nucleus, which is known to involve myosin II (Lehtimäki et al., 2021) by causing excessive ROCK-mediated myosin II activation at this location.

In addition to the stress fibers, Borg5 depletion caused large peripheral ruffles, as revealed by labeling for α-actinin 4 (ACTN4), an actin-crosslinking protein (Fig. 4F). Fluorescent timelapse imaging of actin dynamics with GFP–tagged α-ACTN4 (Movie 1) showed that together this actin organization yielded highly contractile cells in which cell-spanning stress fibers were mechanically linked to the large, dynamic ruffles [Movie 1, KD (+dox)]. In control cells, parallel stress fibers aligning with the long cell axis appeared mechanically unconnected to much smaller ruffles [Movie 1, ctrl (−dox)].

Altered F-actin organization at the level of the basal cortex in Borg5-depleted cells was accompanied by two morphological changes at cell–cell contacting domains. First, by a lack of circumferential cortical F-actin above the plane of attachment (Fig. 4A, compare F-actin in −dox and +dox at 0.5–1.5 µm above the basal domain). Cortical F-actin bundles, which are re-organized from peripheral stress fibers upon cell-cell adhesion, give raise to the MDCK lateral cortex (Rajakylä et al., 2020), which in turn triggers compaction (Adams et al., 1998), an increase in cell height with concomitant decrease in cell area. We determined that when grown as cell islands, Borg5KD cells had indeed a shorter lateral domain (Fig. 4C, height, KD±dox) but a larger footprint (Fig. 4B,C, area/nuclei, KD±dox) than the corresponding control cells. If and how the reduced cortical actin density contributes to the compaction phenotype remains to be established.

**Fig. 5.**
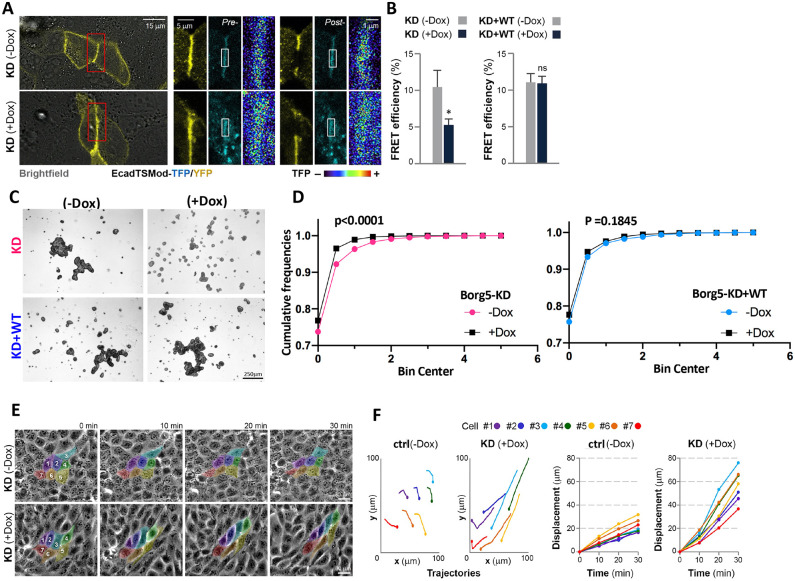
**Borg5-depleted cells have increased E-cadherin-mediated cell–cell adhesion tension, undergo collective streaming and are less resistant to mechanical trituration.** (A,B) FRET efficiency of the E-cadherin tension sensor (EcadTSMod) at cell–cell contacts in Borg5KD or Borg5KD+WT MDCK cells cultured in −dox or +dox conditions. (A) Depicted are examples of pre- and post-bleach images of Borg5KD cells (with and without dox) and the corresponding intensity spectrum maps of the teal fluorescent protein (TFP) donor channel at the regions of interest analyzed (white rectangles). (B) *n*=20 cells/experiment were analyzed for three independent experiments. Error bars show mean±s.d. *P≤0.05; ns, not significant (unpaired two-tailed *t*-test). (C,D) Cell clusters after defined trituration of Borg5KD and Borg5KD+WT spheroid cultures in −dox or +dox conditions. Cells were analyzed from 20 random phase contrast images such as those shown in C, in each of three independent experiments. The cumulative frequency distributions are plotted by area for all objects in D. Cumulative distribution as tested by a Kolmogonov–Smirnov test differed between −dox or +dox conditions for Borg5KD with *P*<0.0001, but not between ±Borg5KD+WT (*P*≈0.1845). (E,F) Phase contrast time lapse (1 frame/min, 30 min) of Borg5KD control (−dox) and Borg5KD (+dox) monolayers that were re-plated at 3×10^4^ cells/cm^2^ and grown for 24 h. (F) *xy*-trajectories and total displacement over time was plotted for a group of 7 cells from each monolayer (colored in the stills). Phase contrast images were taken with a 5× (C) or 10× (E) objective.

**Fig. 6.**
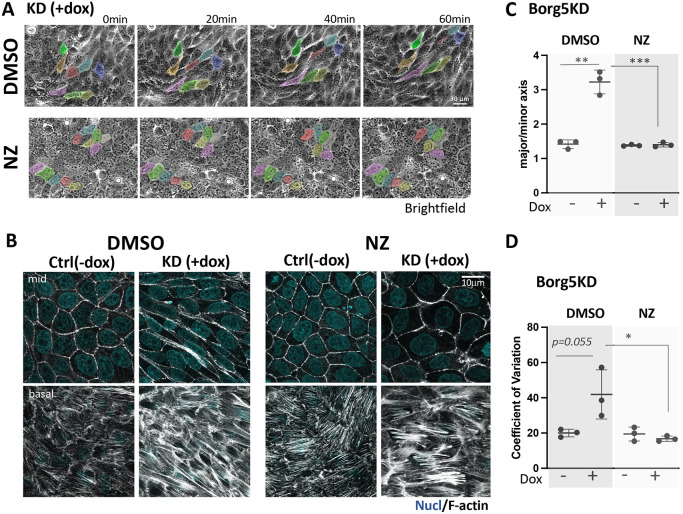
**Nocodazole inhibits Borg5KD induced cell streaming and cell anisotropy.** (A) Stills from a phase contrast time lapse (1 frame/5 min, 60 min with a 10× objective) of induced Borg5KD monolayers that were re-plated at 10^5^ cells/cm^2^, grown for 24 h and treated for 2 h with either DMSO or 10 µM nocodazole (NZ). Note reduced displacement over time of the colored cells in the NZ-compared to the DMSO-treated monolayer. (B) Mid and basal confocal F-actin labeled sections of similarly treated fixed monolayers. Note that NZ prevents stretching of KD+dox cells without reducing their stellate stress fibers. (C,D) Quantification of the ratio major/minor axis (C), and the coefficient of variation of that ratio (D) derived from 10 cells from each of three random images, *N*=3. (C) **P=0.001; ***P=0.0009 (C); *P=0.036 (D) (unpaired two-tailed *t*-test).

Second, Borg5KD cells exhibited higher E-cadherin tension, which we determined using an established FRET-based tension sensor (Borghi et al., 2012) (Fig. 5A,B). This result is consistent with the notion that stress fiber contractility in epithelial cells is counterbalanced by cell–cell adhesion tension (Maruthamuthu et al., 2011). The tension increase upon Borg5 depletion resulted in a modest but significant decrease in cell–cell adhesion (Fig. 5C,D). We deduced the cell–cell adhesion strength from the size of cell aggregates that formed in the absence of cell–matrix adhesion after cells were subjected to defined trituration (Elbert et al., 2006; Foty, 2011). Occasional disruption of cell–cell adhesion combined with migration in confluent Borg5KD monolayers yielded a streaming phenotype (Loza et al., 2016; Sadati et al., 2013) in which groups of highly stretched cells moved in unison, resulting in ‘swirls’ (Fig. 5E,F, KD+dox; Movie 2); in control monolayers of similar density streaming activity was muted by comparison (Fig. 5E,F, KD−dox; Movie 2). The level of cell stretching, an indicator of anisotropic forces in directionally migrating cells, was determined as the ratio of major to minor axis length in fixed cells (Fig. 6B–D); this analysis confirmed reduced cell isotropy (Fig. 6C, compare −dox and +dox in DMSO) and a trend to higher variance in cell shapes (Fig. 6D, compare −dox and +dox in DMSO) in dox-induced Borg5KD cells than in their −dox controls.

Because Borg5 depletion in single cells promoted MT-dependent cell shape changes, we interrogated the role of MTs in the observed streaming phenotype by utilizing nocodazole. Nocodazole treatment reduced streaming (Fig. 6A; Movie 3, compare DMSO and NZ) as well as cell stretching (Fig. 6B,C compare +dox DMSO versus NZ) and the variation in cell shape (Fig. 6D, compare +dox DMSO versus NZ), indicating that these features depend on MT-supported protrusive activities. Nocodazole as well as Rac1 depletion also decreased the ACTN4-positive protrusions (Fig. 4F,H, compare +dox DMSO versus NZ; Fig. S4), indicating that ruffle formation is contingent on MT-dependent Rac1 activation.

MT analysis showed that there were stabilized (i.e. acetylated MTs) at the basal domain in Borg5KD cells that were not seen in controls (Fig. 4D), which was reflected in higher basal acetylated tubulin fluorescence intensity (Fig. 4E, KD±dox). Segments of these MTs aligned with septin 9-positive filaments (Fig. 4D, zoom1, 2), which in Borg5-depleted cells showed little co-occurrence with F-actin. Given that septins can stabilize MTs (Bai et al., 2013; Kuzmić et al., 2022), Borg5 depletion might contribute to MT stability by allowing more extensive septin–MT interactions.

Taken together, Borg5 depletion prior to MDCK monolayer establishment induces changes in F-actin and MT organization that yield highly contractile cells that exhibit migratory behavior in confined spaces.

Co-expression of WT Borg5 with the shRNAmir-construct rescued all Borg5KD phenotypes described above. Thus, WT Borg5 prevented increased acetylated tubulin IF intensity at the basal domain (Fig. 4E, KD+WT ±dox; Fig. S5D), the stellate stress fiber phenotype (Fig. S5A), compromised cell compaction (Fig. 4C, KD+WT±dox; Fig. S5B) and the extensive ACTN4-positive protrusions (Fig. 4G, KD+WT±dox; Fig. S5C). WT Borg5 expression also prevented the tension increase on E-cadherin adhesions (Fig. 5B, KD+WT) and the reduced cell–cell coherence (Fig. 5C,D, KD+WT) observed in Borg5KD monolayers. This indicates not only specificity of the shRNA but also that, unlike what occurs on reduction of Borg5 levels, overexpression of Borg5 had no adverse effect on these aspects of MDCK morphology.

To assess whether Borg5 plays similar roles in other cell types, we analyzed the effects of Borg5 depletion in HeLa cells, where ROCK1 activation, like in MDCK cells, induces stellate stress fibers (Ishizaki et al., 1997). We found that as in MDCK cells, HeLa cell Borg5 colocalized with septin 2 on stress fibers under the nucleus (Fig. S6A) and that the F-actin population there increased upon Borg5 depletion (Fig. S6A,D, yellow arrowheads; nuclear area fraction covered by F-actin quantified in Fig. S6B). Also, similar to what occurred in nonadherent MDCK cells (compare to Fig. 3E), Borg5-depleted HeLa cells frequently lacked compressed circumferential F-actin bundles but instead presented with cortex-parallel F-actin stacks (Fig. S6A,D, blue arrowheads, phenotype frequency quantified in Fig. S6C). By contrast, Borg5 depletion did not cause the septin9–MT alignment (Fig. S6D) or the formation of lamellae or neurite extensions, as seen in MDCK cells, suggesting that the actin, but not the MT-dependent phenotypes are conserved in HeLa cells.

### Borg5 depletion or overexpression causes apical surface deformation in mature monolayers

**Fig. 7.**
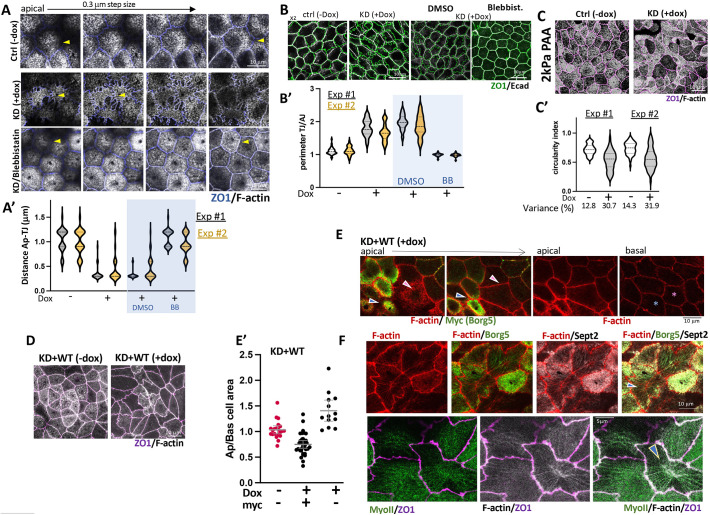
**Borg5 depletion and overexpression in mature monolayers alter apical contractility.** Borg5KD cells (A–C) or Borg5KD+WT cells (D–F) were first cultured in the absence of dox to a density of 1.5×10^5^ cells/cm^2^ on Transwell inserts for 24 h and then for an additional 48 h in either −dox (ctrl) or +dox (KD) (A,B,D–F) or cultured, after dox-pre-induction at a plating density of 10^5^ cells/cm^2^ for 48 h on collagen-coated polyacrylamide hydrogels with an estimated Youngs modulus of 2 kPa (2 kPa PAA) (C). Where indicated, cells were treated for 4 h with 25 µM blebbistatin or DMSO as solvent control. (A,A′) Serial *x-y* confocal sections from the apical (apex) towards the basal domain of cells labeled for ZO1 and F-actin; note that the F-actin-positive apex rises above the TJs in controls cells, but coincides with TJs in the KD cells (arrowheads). Results are quantified in A′ in 30 cells from 0.3 µm *x-y* sections as the distance in µm from the most apical point to the plane of their tight junctions; *N*=2 experiments are presented separately. (B,B′) Overlay of an *x-y* plane at TJs labeled for ZO1 and at adhesion sites labeled for E-cadherin 2 µm below. (B′) The ratios of individual cell perimeters at TJ to that at the AJ was plotted for 30 cells from *N*=2 experiments are presented separately. (C,C′) Merged confocal apical and TJ planes and (C′) circularity index at TJs determined from 50 cells in two separate experiments. Indicated are the data set variance coefficients. Violin plots in A′, B′ and C′ highlight median and quartiles with dashed lines. (D) Merged confocal apical and TJ planes showing irregularly sized and shaped surfaces in Borg5KD+WT cells. (E,E′) Views of apical and basal (1 µm above attachment plane) regions of adjacent cells expressing Borg5 at high (blue arrowhead and asterisk) and low (pink arrowhead and asterisk) levels. (E′) Apical to basal surface area ratio in cells from uninduced control cultures and cells in dox-induced cultures separated into Borg5–Myc expressors and non-expressors; data points are collated from *N*=2 experiments. Error bars shown mean±95% c.i. (F) Apical surface views of depicting Borg5, septin 2 and myosin II (MyoII)-positive F-actin fibers (arrowheads) in irregularly shaped apices. Images in F are representative of two experimental repeats.

When Borg5 depletion was induced after the establishment of polarized mature monolayers, the most conspicuous phenotype were distortions of the apical surface and TJs. These included a high variation in the shape and surface area of apical domains (Fig. 7A–C, KD −dox versus +dox) and frequent fingerlike undulations of ZO1-labeled TJs [Fig. 7A,B, ctrl versus KD; Fig. 7B′, quantified as ratio of perimeters at TJs and adherens junctions (AJs)]. These were accompanied by ‘sunken’ apices in which the top of the apical domain was at level with tight junctions rather than dome-like (Fig. 7A, ctrl versus KD; Fig. 7A′, quantified as distance from apex to TJs). TJ tortuosity results from a difference in forces that neighboring cells exert on shared TJs (Lynn et al., 2020). Myosin II inhibition with blebbistatin eliminated the exaggerated TJ tortuosity (Fig. 7B,B′, compare KD with and without blebbistatin) and restored a dome-like appearance to the apical domain (Fig. 7A,A′, compare KD with and without blebbistatin), indicating that high myosin contractility of the mechanically coupled TJs and apical domains caused both phenomena. The apparent apical force imbalances are unrelated to stress fiber formation as we observed a similar phenomenon when cells were plated on a soft matrix (collagen-functionalized polyacrylamide gels of 2 kPa) where stress fiber formation did not occur (Fig. 7C, 2 kPa PAA). It manifested as high variability in apical cell shape as measured by the circularity index (Fig. 7C′).

Rescue experiments with recombinant Borg5 revealed that elevated Borg5 likewise distorted the cell apex (Fig. 7D). High apical Borg5 levels were associated with small apical surfaces in comparison to neighbors expressing lower levels of Borg5 (Fig. 7E, blue versus pink arrowheads) without distorting cell shape along the basolateral aspect of the cells (Fig. 7E, blue versus pink asterisks), resulting in different apical/basal surface ratios for high and low/no Borg5–Myc-expressing cells in the same monolayer (Fig. 7E′). Borg5 and co-recruited Septin2 could be seen localized around F-actin fibers spanning across the apical surface, often emanating from the apical median, and apparently pulling on the apical cortex (Fig. 7F). These unusual apical F-actin fibers are likely the direct result of the exaggerated Borg5 and septin levels.

Thus, F-actin organization at the basal domain and restriction of cell–cell adhesion tension requires a minimal level of Borg5 and is insensitive to Borg5 overexpression, whereas apical tension homeostasis is sensitive to both reduced and elevated Borg5 levels.

### Borg5 interacts with the septin-binding rod-domain of myosin IIA

**Fig. 8.**
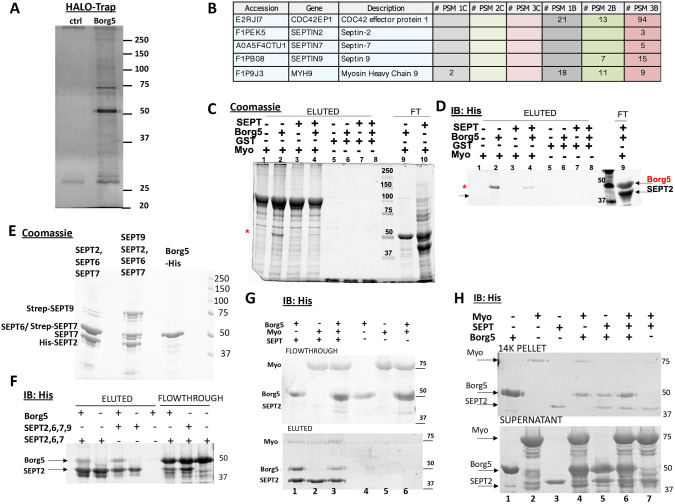
**Septins compete with Borg5 for the binding of the MyoIIA-Rod domain.** (A,B) Silver-stained gel of HALO-Trap isolated proteins from mock (ctrl) and Borg5–HALO transfected MDCK cells, showing Borg5-specific co-isolation of cellular proteins (A), and number of spectral peptide matches (#PSM) from Borg5, septins and MyoIIA isolated from control (1-3C) and Borg5–HALO (1-3W) samples in three experiments (B). (C,D) Coomassie gel (C) and His blot (D) of a His–Borg5 binding assay to glutathione–agarose immobilized GST-tagged MyoII-Rod domain or to GST in the presence or absence of SEPT complexes (His–Sept2/6/7/9–Streptag). Flowthroughs (FT) and SDS eluants are shown. SEPT complexes reduced His–Borg5 binding to Myo–GST to 0.6 (experiment 1) and 0.3 (experiment 2) of that observed without septins present. Note that binding of His–SEPT2 to Myo–GST (lanes 3,4) is undetectable. Asterisks indicate position of His–Borg5; arrow in His-IB indicates position of SEPT2. (E,F) Recombinant His–Borg5 bound to Streptag-immobilized His–SEPT2/6/7–Strep and to His–SEPT2/6/7/9–Strep. Shown are Coomassie stains of the inputs (E) and His immunoblots, revealing both His–Borg at 50 kDa and His–SEPT2 at ∼42 kDa, in the flowthrough and biotin-eluted fractions (F). (G) His IB of flowthrough and biotin eluant of a His–Borg5 binding assay to Streptag immobilized His–SEPT2/6/7/9 (SEPT) in the presence or absence of the His-tagged Rod domain of MyoIIA (Myo). Asterisk indicated the position of Borg5 in the eluant. In four experiments Borg5–SEPT binding in the presence of His-MyoIIRod was 0.57±0.19 (mean±s.d.) of that without MyoIIRod. Note that unlike Borg5, Myo is largely restricted to the flowthrough. (H) Incubation of His–Borg5 with His-MyoRod and/or SEPT complexes (SEPT2/6/7/9) reduces Borg5 presence in pellets from a 14,000 ***g*** centrifugation. Positions of His–MyoRod, His–SEPT2 and His–Borg5 in the His IB of supernatant and pellet are indicated. Note that the reduction of pelleted Borg5 in lanes 4, 5 and 6 compared to lane 1. Images shown are representative of *N*=2 (C–F,H), *N*=3 (A) or *N*=4 (G).

Borg proteins are thought to promote septin interactions with F-actin, which in turn facilitates actin polymerization and/or bundling (Lam and Calvo, 2019; Tomasso and Padrick, 2023). Our findings suggest a different function for Borg5 in MDCK cells as Borg5 overexpression did not increase basal stress fiber levels; instead, stress fiber formation at sites of Borg–septin colocalization was induced upon Borg5 depletion. This prompted us to investigate whether Borg5 has functional partners that might inhibit or modulate the effects of septins on F-actin. To that end, we performed mass spectrometry analysis of proteins that specifically co-isolated with HALO-tagged Borg5 from MDCK Tx100 lysates (Fig. 8A). The results from three independent experiments revealed several highly abundant proteins and a larger number of less abundant putative binding partners, isolated with fewer peptide spectral matches (#PSM) (Table S1). Septins (septin 9>7>2 in order of #PSM) were among the low abundant interactors (Fig. 8B); thus, the robustness of Borg5 co-isolation might not correlate with the extent of the interactions *in vivo*. We speculate that because Borg5 is predicted to be largely disordered, pH and ionic strength of the lysis buffer and exposure to proteins that Borg5 does not see in intact cells, might all influence the *in vitro* Borg5 interactome.

Among the putative Borg5 interactors we selected myosin IIA (MyoIIA, herein referring to the MYH9 heavy chain) for further investigation because myosin activity was associated with each of our identified Borg5-regulated processes and because septins have been reported to bind and regulate myosin II function.

We established, using recombinant proteins in cell-free assays, that His–Borg5 interacted with the GST-tagged rod domain of MyoIIA (MyoIIA-Rod) directly (Fig. 8C,D, compare lanes 1 and 2, in Coomassie and His-IB; red asterisks indicate migration of Borg5). Given that the MyoIIA rod domain is also a reported septin-binding partner (Joo et al., 2007), we further investigated whether septins modified the MyoIIA-Rod interaction with Borg5. To that end, we first confirmed that recombinant His-tagged Borg5 bound to immobilized septin 2–septin 6–septin 7 (hereafter septin 2-6-7) or septin 9–septin 6–septin 7–septin 2 (hereafter septin 9-6-7-2) complexes (Fig. 8F). These complexes were prepared according to Castro-Linares et al., (2022) by bacterial co-expression of 6×His-tagged septin 2 with either untagged septin 6 and Strep-tagged septin 7, or with septin 6, septin 7 and Strep-tagged Septin 9, and their sequential purification first on nickel followed by Streptag resin (Fig. 8E). We then included septin 9-6-7-2 complexes in three-way binding assays. Pre-incubation of Borg5 with septin complexes reduced the recruitment of recombinant His–Borg5 to immobilized GST–MyoIIA-Rod (Fig. 8C,D, asterisk, compare lanes 2 and 4 in Coomassie gels and His blots, respectively). Conversely, pre-incubation of His-tagged MyoIIA-Rod with His–Borg5 diminished Borg5 binding to immobilized septin complexes (Fig. 8G, compare Borg5 in lanes 1 and 3 in eluted fractions). Collectively these findings indicate that septins and MyoIIA-Rod hinder the interactions of each other with Borg5. Septin or MyoIIA-Rod interaction with His–Borg5 also reduced the tendency of Borg5 to appear in low-speed (14,000 ***g***) pellets (Fig. 8H, compare Borg5 in lane 1 to that in lanes 4–6), likely by stabilizing a conformation that prevents Borg5 aggregation or multimerization. Although we were unable to determine whether Borg5 in turn interfered with the reported septin–MyoII interaction, because under the salt conditions (300 mM) required to minimize His–Borg5 aggregation, MyoIIA-Rod binding to septins was weak (Fig. 5D, note lack of visible His-Sept2 (black arrow) in lanes 3,4 of His-IB) and the effect of Borg5 on MyoIIA-Rod–septin interaction is too inconsistent to allow a conclusion, we speculate that Borg5 finetunes the reported septin-mediated myosin II activation. As Borg5 depletion in contacting cells increased stress fiber-associated ROCK-dependent myosin activity (Fig. S2B), Borg5 might competitively interfere with myosin–septin binding directly or with the reported recruitment of MyoIIA-activating proteins, including ROCK, to septin scaffolds (Joo et al., 2007).

## DISCUSSION

Our findings reveal that, in polarized MDCK cell monolayers, Borg5 prevents excessive contractility, surface anisotropy and migration. We attribute this role to the combined effects of three separable functions, as below.

First, Borg5 depletion induces formations of a septin population that is distinct from F-actin trajectories and which overlapped with MTs. Although in polarized cells, few MTs run parallel to the basal domain, we observed that Borg5KD cells had an increased basal population of acetylated tubulin, segments of which colocalized with septins; this population of stable MTs could arise by MT plus end capture at the cortex, as plus-end capture stabilizes MTs (Kaverina et al., 1998). Based on these findings, we propose a model in which Borg5 restricts the reported septin-dependent MT plus end targeting into the cortex (Bowen et al., 2011) where MTs, through their known role in sequestering the myosin II-activating GEF-H1, antagonize the formation of circumferential ventral stress fibers and promote Rac1-mediated lamellae (Even-Ram et al., 2007; Rafiq et al., 2019; Seetharaman and Etienne-Manneville, 2019). Borg5 depletion in MDCK cells might thus have a similar effect – promoting septin association with MTs rather than with F-actin – as has been associated with the downregulation of Borg2 and Borg3 upon Taxol-mediated MT stabilization (Salameh et al., 2021).

Second, independent of its effect on MTs, Borg5 depletion causes the formation of ROCK-dependent stellate stress fibers with a myosin II-enriched hub under the nucleus and focal adhesion anchorages at the cell periphery. Acto-myosin asters resembling this organization form transiently during developmental processes *in vivo* (Blanchard et al., 2010; Munro et al., 2004; Vasquez et al., 2014) where they have been associated with cortical contractile myosin pulses (Martin, 2020). Given that Borg5 depletion yielded stellate stress fibers that were stable, it might be worthwhile investigating whether septins and Borg proteins contribute to their oscillatory behavior in vertebrate morphogenesis. Myosin accumulation in the center of asters in Borg5-depleted MDCK cells might deplete myosin elsewhere and prevent myosin II-dependent F-actin remodeling into lateral cortical F-actin bundles, thereby exacerbating the MT-dependent myosin II inhibition at the periphery discussed above. Lack of peripheral F-actin bundles in turn might favor lamellae and inhibit the development of the lateral domain and cell height.

Finally, Borg 5 also regulated myosin-dependent contractility at the apical domain where Borg5 was enriched in compacted MDCK monolayers. Here, Borg5 was crucial for the equalized apical force balance that ensures isotropic shape and size of the TJ-delineated apical domains of cells within the monolayer. Apical forces are transmitted between cells via contractile F-actin associated with their apical junctional complexes (AJCs) (Citi, 2019). These complexes are in turn mechanically coupled to the cytoskeleton forming the apical cortex, which in MDCK cells consists of an isotropic interconnected network of actin and myosin filaments (Klingner et al., 2014). Apical shape changes can be due to altered forces at TJs, as has been shown experimentally by altering their composition (Odenwald et al., 2018; Tokuda et al., 2014; Brückner and Janshoff, 2018; Hatte et al., 2018; Martínez-Ara et al., 2022; Rouaud et al., 2023), or it can be triggered by changes in contractility of the apical cortex itself, as during apical constriction in gastrulation (Martin, 2020; Vasquez et al., 2014). Given that Borg5 does not localize to TJs but to the apical cortex, it likely regulates contractility there. We expect the underlying mechanisms of action to be different from those operating at the basal domain, because increased apical Borg5 levels upon overexpression caused *de novo* formation of apical radial F-actin fibers, whereas increased Borg5 levels at ventral stress fibers did not alter stress fibers there.

Beyond differences in F-actin regulation at apical and basal domains of MDCK cells, the net effect of Borg5 loss of function also differs between cell types. Thus, we found that in HeLa cells, Borg5 depletion promoted subnuclear stress fibers but not the MT-dependent lamellae; furthermore, an inhibition of directional vascular endothelial cell migration reported in Borg5 knockout mice was associated with an apparent decrease, rather than increase, in cellular myosin II activity (Liu et al., 2014). Among cell type-specific differences expected to modify phenotypic outcomes are: (1) the expression profile of other Borg family proteins, which, in the absence of Borg5, might execute alternative septin-mediated cytoskeletal organization; (ii) the abundance of the Septin 9_i1 splice variant, which mediates septin alignment with MTs in the absence of Borg proteins (Kuzmić et al., 2022); and (iii) the constellation of available Borg5-binding partners in each cellular context. Borg5 is predicted to be highly disordered, which likely explains the large number of interaction partners we co-isolated with Borg5 from cell lysates and which poises Borg5 to a engage in a wide range of low-affinity interactions. This in turn is a feature well suited to either ‘toning’ or implementing several alternative scaffolding functions of septins.

Given the central place myosin II occupies in Borg5-dependent F-actin organization, we believe that the direct interaction of Borg5 with the septin 2-binding rod-domain of myosin IIA and the competition of septins for this interaction, which we established in cell-free assays, is a crucial part of the cellular role of Borg5. Septin 2 has been shown to recruit myosin IIA to actin filaments and might scaffold ROCK proteins to mediate myosin II activation (Joo et al., 2007). Competition for Borg5 binding could release myosin IIA from this scaffold, thereby limiting septin-mediated myosin II activation, a scenario consistent with increased myosin II activity in Borg5-depleted MDCK cells. However, our Borg5 interactome also included several RhoGEFs (including ARHGEF2) as well as the actin-crosslinking proteins α-actinin 1, α-actinin 4 and filamin A, which if recruited to septin-aligned F-actin filaments, could stimulate Rho-ROCK-mediated myosin activation or F-actin bundling, respectively, a scenario more consistent with previously reported Borg functions in other contexts. The challenge ahead is thus to characterize unique Borg5-containing protein complexes that are associated with specific phenotypes.

## MATERIALS AND METHODS

### Cell culture and protein expression and depletion

MDCK and HeLa cells were obtained from Enrique Rodriguez-Boulan (Weill Cornell Medical College, New York, USA) and maintained below confluence in DMEM (Corning #10-013-CV) with 10% fetal calf serum at 37°C in a 5% CO_2_ humidified atmosphere. Cells were yearly tested for mycoplasm but were not independently authenticated. For experimental analyses, cells were cultured on plastic (for biochemical experiments), MatTek glass bottom dishes (P35G-1.5-14-C; MatTek Corporation) (for time lapse imaging), or on glass coverslips (CLS-1760-012; Chemglass), 0.4 µm pore Clear Polyester Transwells (#3460, Corning) or on polyacrylamide hydrogels (for IF analysis). Induction of the dox-inducible expression cassettes in MDCK cells occurred by pre-treatment with 1 µg/ml dox (doxycycline hyclate; Sigma Millipore #D-09891) for 48 h prior to re-plating at a density of either 10^5^ or 3×10^5^ cells/cm^2^ and continuously cultured in the presence of 1 µg/ml dox for 24 h. For single-cell analysis, cells were re-plated after 72 h of induction at 2.5×10^3^ cells/cm^2^ for 3 h.

For canine Borg5 KD, two target sequences were used [5′-CGGTGAGCAAGTTCACCTTTGA-3′ (#1) and 5′-CCGTGACCGAGACCATGATAGT-3′ (#2)]; each yielded a 70–80% reduction in Borg5 protein levels when expressed as shRNAmirs in the dox-inducible pSLIK lentivirus expression system (Addgene #25737; Shin et al., 2006). We utilized #1 for all experiments presented but observed similar morphological changes with #2. All recombinant Borg5 constructs were based on mouse Borg5–Myc cDNA as used previously (Vong et al., 2010) and obtained from the authors. We co-expressed Borg5–Myc together with the shRNAmir #1 from a single dox-dependent transcript in the pEN_TTmiRc2 vector of the pSLIK lentivirus expression system. We transduced MDCK cells with lentiviruses generated with the corresponding pSLIK-Hygro plasmids and selected pools and clones of Hygromycin-resistant cells. His–Borg and HALO-tagged WT Borg5 were generated by PCR cloning into vectors pet28a+ (Novagen) and pFC14K (Promega), respectively. In the pFC14K vector, the C-terminal HALO tag is separated from Borg5 by a TEV protease cleavage site.

HeLa cells were reverse transfected with a combination of two Borg5 siRNAs (Qiagen) using RNAiMax (Invitrogen) according to the manufacturer's instructions; after 48 h cells, were re-plated onto coverslips coated with 10 µg/cm^2^ rat tail collagen I (Corning, # 354236) at a density of 20,000 cells/cm^2^ and cultured overnight. The Borg5 target sequences were: 5′-CACGGACGGCCACTCCAGCTA-3′ and 5′-TTCTCTGCGCTTGAACATCTA-3′.

cDNAs of EcadTSMod TFP/YFP (Borghi et al., 2012), provided by Nicolas Borghi (Institute Monod, Paris, France), MyoIIA-pEGFPC3, provided by Anne Bresnick, Albert-Einstein College of Medicine, Bronx, USA, ACTN4-pEGFPN1 (Shao et al., 2010), provided by Alan Wells, University of Pittsburgh, USA were introduced by transient transfection with AMAXA nucleofector II (Lonza) and analyzed 24 h upon transfection. Rac1 siRNA (sequence, 5′-UUUACCUACAGCUCCGUCUCCCACC-3′, custom synthesized by Sigma Millipore) is based on a previously published target sequence (Nakahara et al., 2015) and was introduced by reverse transfection with RNAiMax (Invitrogen) according to the manufacturer's instructions. Allstars negative control (Qiagen #1027281) was used as RNAi control. Transfected cells were dox-induced and cultured for 48 h before being re-plated at the desired cell density for an additional 24 h prior to analysis.

ML-7, Y27632, blebbistatin and nocodazole (Cayman Biologicals) were added from freshly made 1000× stocks in DMSO.

### Preparation of hydrogels

Fabrication of 2 kPa collagen-functionalized polyacrylamide (PAA) gels was undertaken according to the detailed protocol previously provided (Tse and Engler, 2010). Briefly, gels were polymerized on amino-silanated 12 mm circular coverslip(s) by lowering them onto 25 µl drops of a PAA mix placed on chloro-silanated glass slide(s). For an estimated elasticity modulus of 2 kPa, gels of 4% acrylamide and 0.1% Bis-acrylamide were polymerized with a 1/1000 volume of each tetramethylethylenediamine (TEMED) and 10% (w/v) ammonium persulfate (APS). Amino-silanation of the coverslips was by sequentially covering them with 0.1 M NaOH, which was heat evaporated, and reacting with 3-aminopropyltriethoxysilane (APES) and, after extensive rinses, crosslinking with 0.5% glutaraldehyde. Chloro-silanated glass slide(s) were prepared by spreading them with dichlorodimethylsilane (DCDMS), wiping off the excess coating and rinsing them with double-distilled (dd)H_2_O. Collagen I-functionalization of the PAA gels occurred by UV crosslinking 0.2 mg/ml sufosuccinimidyl-6-(4′-azido-2′-nitrophenylamino)-hexanoate (sulfo-SANPAH; Pierce Biotechnology) with a UV 360 nm lamp at a distance of 3 inches for 10 min onto the PAA and subsequently reacting the crosslinker with 20 µg/cm^2^ rat tail collagen I (Corning, #354236) in 50 mM HEPES, pH 8.5 overnight at 37°C. Coverslips were sterilized by UV irradiation at 254 nm in a UV-Stratalinker 2400 prior to use.

### Cell dissociation assay

For the cell dissociation assay, 10^4^ cells (Borg5KD in −dox or precultured in +dox) were cultured overnight in DMEM with 0.1% serum (to prevent proliferation) in polyHEMA coated 96-U-well plates with gentle rocking. For coating, 50 µl of a 2% polyHEMA (poly 2-hydroxyethyl methacrylate; Sigma cat. #P3932) solution in ethanol was dried overnight in the well under rocking at 37°C. Cell spheroids were triturated by five passages up and down a P200 tip set at 180 µl and transferred in 25 µl aliquots onto glass slides, covered with a coverslip. Random pictures of the cell aggregates were taken by phase contrast with a 5× objective lens with an Axiovert 200 M microscope (Carl Zeiss, Oberkochen, Germany).

### Immunofluorescence, widefield and confocal microcopy

Cells were fixed in either ice-cold methanol (ACTN4, β-actin) or with 4% paraformaldehyde (PFA; all other antigens) at room temperature for 15 min and quenched with 50 mM NH_4_Cl in PBS. For colabeling of tubulin and F-actin with septin 2, cells were fixed in 0.25% glutaraldehyde, 4% PFA, 0.1% Triton X-100, 0.6 U/ml CF 647–phalloidin (Biotum, #00041) in PHEM buffer (60 mM PIPES, 25 mM HEPES, 10 mM EGTA, 2 mM MgCl_2_ pH ∼7.0) and quenched in 10 mg/ml borohydride in PBS. PFA-fixed samples were permeabilized with 0.2% Triton X-100. Blocking and antibody incubation was in 10% FCS and 1% BSA. The following primary antibodies were used for immunofluorescence (IF) and/or immunoblotting (IB): Cdc42EP1/Borg5 (Proteintech, 27904-1-AP, IB 1:500, IF 1:100); Myc tag (clone 9E10, home-made, IB and IF 1:500) or c-Myc tag (Proteintech #16286-1-AP, IF 1:250, IB 1:1000); Vinculin (Novus Biologicals, #NB120-11193, IF 1:100 or Proteintech 26520-1-AP, IF 1:600), ZO1 (clone R26.4C; Stevenson et al., 1986, 1:50 IF); septin 2 (Proteintech #11397-1-AP, IF 1:300 and #60075-1-Ig, IB 1:1000; BiCell #0022, IF 1:100) Septin 9 (Proteintech # 10769-1-AP, IF 1:300), pThr18/pSer19-MLC2 (Cell Signaling #3674, IF 1:200), E-cadherin (clone RR1, deposited by Barry Gumbiner, University Virginia, with DSHB, Antibody Registry # AB_528114, IF 1:10), tubulin (clone YL1/2, Novus Biologicals, #NB600, IF 1:500), acetylated tubulin clone [6-11B-1] (Millipore Sigma #T6793 IF 1:200), HALO-tag (Promega #g928a, IB 1:500), β-actin [2D4H5] (Proteintech #66009-1-IG, IF: 1:500, IB 1:5000), Actinin α4 (ACTN4; Proteintech 19096-1-AP, IF 1:250), GAPDH [clone1E6D9] (Proteintech # 60004-1-Ig, IB 1:2000), 6xHis-tag [clone 1B7G5] (Proteintech # 66005-1-IG, IB 1:5000), Rac1 (BD Bioscience #610650, IB 1:500), and phospho-tyrosine ([clone PY20], Santa Cruz Biotechnology, sc508, IF 1:200).

Primary antibody labeling was at 4°C overnight (for all phospho-antigens) and for 1 h at room temperature for all other antigens. DAPI (Sigma #D8417), Atto 647N–Phalloidin (Sigma #65906) and the following F(ab′)_2_ fragment secondary antibodies from Jackson ImmunoReseach Laboratory were incubated at a dilution of 1:500 for 1 h at room temperature: Rhodamine Red™-X (RRX) AffiniPure™ F(ab′)₂ fragment donkey anti-mouse-IgG (H+L) # 715-296-151; Alexa Fluor^®^ 488 AffiniPure™ F(ab′)_2_ fragment donkey anti-rabbit-IgG (H+L) # 711-546-152; Alexa Fluor^®^ 647 AffiniPure™ F(ab′)₂ fragment donkey anti-rat-IgG (H+L) # 712-606-153.

Fixed cells were imaged by confocal microscopy on a TCS SP5 confocal microscope (Leica Microsystems, Wetzlar, Germany) using a HCX PL APO 40×/1.25–0.75 oil CS objective or an HCX PL APO 63×/1.4-0.60 oil λ_BL_ CS objective on glass coverslips mounted in nonhardening, glycerol-based aqueous mounting medium (DABCO). Confocal (pinhole, 1 Airy Unit; pixel size, 160.5 nm) *x-y-z* and *x-z-y* stacks were taken at a stepsize of 0.3–1 µm. Images were processed with LAS AF v.2.6.0.7266 (Leica Microsystems), ImageJ (Fiji, version 2.1.0/1.53c) or v.1.52i (National Institutes of Health) and Adobe CS6 (Adobe Inc.) software. Where indicated in the figure legends, a subset of planes was merged using the *Z*-projection function with maximal intensity; 3D projections were generated with the ‘3D projection’ function, using the brightest point method, with 1 µm slice spacing and 10° angle rotation with interpolation.

Confocal live-cell imaging was conducted with the HCX PL APO 63×/1.4-0.60 oil λ_BL_ CS objective on MatTek chambers at 37°C in a CO_2_-enriched environmental chamber in growth medium without Phenol Red. Images were collected every 30 s. Image stacks were processed in ImageJ by applying a 1-pixel Gaussian blur, a background subtraction and contrast adjustment.

Phase-contrast imaging was performed on an Axiovert 200 M microscope (Carl Zeiss, Oberkochen, Germany). Cells were imaged using an EC Plan-Neofluar 10×/0.30 Ph 1 objective on MatTek chambers at 37°C in a CO_2_-enriched atmosphere in growth medium without Phenol Red. Cells in Fig. 2C were imaged with an EC Plan-Neofluar 5×/0.15 Ph 1 objective. Images were acquired with a Hamamatsu ORCA-R^2^ cooled-CCD camera controlled with AxioVision v4.8.1.0 (Carl Zeiss) software. For time-lapse experiments, images were collected every 5 min, using an exposure time of 100 ms and 1×1 camera binning.

#### FRET analysis

Förster resonance energy transfer (FRET) upon transfection of the EcadTSMod TFP/YFP was measured in live cells. FRET efficiency was calculated with the acceptor-bleaching FRET module (FRET-AB) of the LAS AF software. For the setup, an HCX PL APO 40×/1.25-0.75 oil CS objective was used to obtain pre- and post-bleaching confocal (pinhole, 1 AU; pixel size, 63.1 nm; line average, 4) *x-y* sections. For the bleaching, a region of interest at the cell–cell contact was exposed to the 488 argon laser (main power; 80% with 514 nm laser line; 100%, for 10 iterations). The FRET efficiency was evaluated only in regions of interest with at least 75% of reduced fluorescence intensity in the acceptor species.

### Quantitative analysis of morphology and protein distribution

#### Fluorescence intensity measurements

Integrated density fluorescence was determined in ImageJ from individual *x-y* image planes with a set threshold after subtracting background fluorescence deduced from unlabeled image planes. For comparison between experiments, values were normalized to the lowest measured value in a dataset.

#### Correlation between Borg5 and stress fiber intensities

For each data point, the average Borg5 fluorescence intensity along a line crossing F-actin filaments under the nucleus was plotted against the average F-actin peak intensity along that line. Peak intensities were recorded from Image J Profile Plots. For normalization, the lowest intensity from each data set was set to 1.

#### Cell displacement in confined monolayers

Individual adjacent cells were tracked with the manual option of TrackMate v6.0.0 by following a nucleolar mark in each of the 30 frames. *x-y* coordinates and pixel distance were exported to PrismGraph v.7.0 software for documentation. Pixel size values (0.645 µm for 10× and 1.29 µm for 5× lens) were calibrated to actual distance.

### Recombinant protein purification

BL21(DE3) cells (New England Biolabs #C2527I) were transformed with either His–Borg5 in vector pet28a, His–MyoIIA-Rod domain (aminos acids 1339–1960) in pet28a and provided by Anne Bresnick, or GST-MyoIIA-Rod, which is the same MyoII fragment cloned into in pGEX4T-3 vector (Cytiva GE28-9545-521). For septin complex isolation, pnEA-vH_His-TEV-Sept2 (Addgene #174491) was co-tranformed with pnCS_SEPT6_SEPT7-TEV-Strep (Addgene #174499) or pnEA-vH_His-TEV-SEP2_SEP6 (Addgene #174497) was transformed together with pnCS_SEPT7_SEPT9_i1-TEV-Strep (Addgene #174500).

Cultures were grown in Terrific Broth (12 g/l tryptone, 24 g/l yeast extract, 4 ml/l glycerol, 17 mM KH_2_PO_4_, 72 mM K_2_HPO_4_) until OD_260_ 2-3, and recombinant protein expression induced with 1 mM IPTG for 3 h at 30°C; cell pellets were stored frozen at −20°C until processing as below.

For His–Borg5, cells were thawed and resuspended on ice in PBS, boiled for 10 min and then plunged into an ice-water bath for 10 min. The 20,000 ***g*** pellet fraction was resuspended in fresh 8 M urea buffer containing 10 mM Tris-HCl, 500 mM KCl and10 mM imidazole (pH 7.5) and bound to Ni-NTA agarose (Invitrogen, #R90115) in batch, washed in binding buffer, followed by binding buffer in the absence of urea and eluted with 1.5 bed volume of 500 mM imidazole,10 mM Tris-HCl pH 7.5, 2 mM MgCl_2_ and 300 mM KCl. Protein containing fractions were pooled and dialyzed against 10 mM Tris-HCl pH 7.5, 2 mM MgCl_2_ and 300 mM KCl and snap-frozen in aliquots. We also purified His–Borg5 under native conditions as described for His–MyoII-Rod and septins below; proteins from both preparation methods bound septin complexes equally, but when prepared under native conditions, more degradation occurred, and even with an Mg-ATP incubation step, heat-shock proteins co-purified.

Septin complexes were isolated as described previously (Castro-Linares et al., 2022). Briefly, cells were resuspended in septin buffer (10 mM Tris-HCl, 2 mM MgCl_2_, 300 mM KCl) supplemented with 10 mM imidazole, 10 mM Mg_2_SO_4_, 1 mM DTT, benzonase (Sigma# E8263-5KU) 0.1 µl/ml buffer, EDTA-free protease inhibitor cocktail (MedChem Express #HY-K0011) and 1 mg/ml lysozyme (Sigma Millipore #L-6876). Lysis was by sonication and subsequent addition of 0.5% Triton X-100. Cleared lysates were incubated with nickel agarose, washed with 10 column volumes of septin buffer with 20 mM imidazole and eluted with 500 mM imidazole in septin buffer. Fractions were pooled according to protein content, dialyzed against septin buffer with 1 mM DTT and snap-frozen in single-use aliquots. For Borg5-Septin binding assays, septin aliquots were column-loaded onto Strep-Tactin^®^ Sepharose^®^ (IBA, #2-1201-002), washed with septin buffer and eluted with 5 mM D-biotin in septin buffer.

His–MyoIIRod was purified according to the septin protocol except that, to remove associated heat-shock proteins, the lysate was incubated with 2 mM Mg-ATP for 10 min at 37°C prior to nickel agarose binding; GST–MyoIIRod was prepared similarly, except for omission of imidazole in the buffers and binding to glutathione–Sepharose 4B-CL (Bioworld #20181088-1) and storing the isolated protein on the resin in septin buffer with sodium azide for up to 14 days.

### Recombinant protein binding assays

Protein concentrations were estimated from Coomassie-stained gels and proteins were combined in equimolar ratios. Incubations were in septin buffer, supplemented with 1 mM DTT and 0.05% Tween 20 at 4°C for 90 min, including batch-binding to glutathione–Sepharose- and Streptag–Sepharose-immobilized proteins. After binding, Sepharose resins were washed four times in septin buffer and elution was with 5 mM D-biotin (Acros Organic, AC230090010) for Streptag-Sepharose binding and with SDS-PAGE buffer for glutathione–Sepharose binding.

### Immunoblotting

PAGE gels were transferred onto Immobilon-FL membrane (Millipore) and blocked with 5% milk; primary antibody incubation was in 1% BSA and 1% fish serum or SEA BLOCK (ND-R0999 Novatein Biosciences), DyLight 680- or 800-coupled secondary antibodies were incubated in 5% milk. Blots were imaged with a Laser scanner Fuji Typhoon NIR Plus, (Amersham) or LiCor Odyssee (LiCor) and analyzed/quantified with ImageQuant (Amersham) or LiCor Image Studio Lite software. Loading was controlled by GAPDH. The raw scanned blots as well as full sized Coomassie gels are shown in Fig. S7.

### HALO-trap isolation of Borg5 complexes from MDCK cells

2×10 cm dishes of confluent MDCK cells, transiently transfected with Borg5-TEV-HALO or control mock transfected cells, were lysed in 10 mM Tris-HCl pH 7.5, 150 mM NaCl, 0.5 mM EDTA, 1 mM DTT, 0.5% Triton C-100 and Protease Inhibitor Cocktail (Promega G6521). DNA was sheared by passing lysate through a 30 g needle and insoluble material removed by a 20 min 20,000 ***g*** centrifugation. Cleared lysate was incubated for 2 h with end-over-end rotation at 4°C with HALO-Trap agarose (Chromotech/Proteintech, #ota), the HALO resin was washed fours time with lysis buffer and incubated with 1 µl His-TEV protease (Genscript# C744N34) in 50 mM Tris-HCl and 100 mM NaCl pH 8.0 plus 5 mM DTT at 4°C overnight to release Borg5 from the HALO moiety. The supernatant and two washes (without DTT) were twice passed over a nickel-agarose spin column to remove TEV protease, precipitated and solubilized in SDS sample buffer.

### Mass spectrometry

#### Preparation of samples for mass spectrometry

Proteins were reduced with 2 µl of 0.2 M dithiothreitol (Sigma) for 1 h at 57°C. Samples were cooled to room temperature and then alkylated with 2 µl of 0.5 M iodoacetamide for 45 min at room temperature in the dark. NuPAGE LDS Sample Buffer (1×) (Invitrogen) was added to the samples and the samples loaded onto a NuPAGE^®^ 4-12% Bis-Tris Gel 1.0 mm (Life Technologies). The samples were run until just passed the stacking region to remove any LCMS incompatible reagents. The gel was then run for 20 min at 200 V, stained with GelCode Blue Stain Reagent (Thermo Scientific) and the entire gel lane excised and destained with 1:1 (v/v) methanol and 100 mM ammonium bicarbonate. Gel pieces were partially dehydrated with acetonitrile then further dehydrated using a SpeedVac concentrator. For proteolytic digestion 300 ng of trypsin (modified, Promega) was added, followed by 200 µl of 100 mM ammonium bicarbonate. The digestion was allowed to proceed overnight at room temperature with light agitation. To stop the digestion and assist with peptide extraction a solution of 5% formic acid in acetonitrile 1:2 (v:v) was added and incubated for 15 min with agitation. The solution was transferred into a new tube and the procedure repeated two more times. The combined solutions were dried down in a SpeedVac concentrator to remove the acetonitrile. The samples resuspended in 0.1% acetic acid and loaded onto equilibrated Ultra-Micro SpinColumns™ (Harvard Apparatus) using a microcentrifuge. The spin columns were washed three times with 0.1% trifluoroacetic acid and the last wash with 0.5% acetic acid. Peptides were eluted with 40% acetonitrile in 0.5% acetic acid, followed by 80% acetonitrile in 0.5% acetic acid. The solutions combined and dried down using a SpeedVac concentrator. The samples were reconstituted in 0.5% acetic acid and stored at −80°C until analysis.

#### Mass spectrometry analysis

An aliquot of each sample was LC separated on an Easy-nLC 1200 HPLC (Thermo Fisher Scientific) by loading it onto an Acclaim PepMap trap column (2 cm×75 µm) in-line with an EASY-Spray analytical column (50 cm×75 µm ID PepMap C18, 2 μm bead size). The sample was gradient eluted into a Thermo Fisher Scientific Orbitrap Eclipse Tribrid Mass Spectrometer using the following gradient: in 5 min to 5%, 60 min to 35%, 10 min to 45% and another 10 min to 100% solvent B and hold at 100% B for 10 min (solvent A, 2% acetonitrile in 0.5% acetic acid; solvent B, 80% acetonitrile in 0.5% acetic acid). The flow rate was set to 200 nl/min. High resolution full mass spectrometry (MS) spectra were acquired with a resolution of 240,000, an automatic gain control (AGC) target of 10^6^, a maximum ion time of 50 ms, and a scan range of 400–1500 *m*/*z*. All MS/MS spectra were collected using the ion trap in rapid scan mode with an AGC target of 2×10^4^, maximum ion time of 18 ms, one microscan, 0.7 *m*/*z* isolation window, and a normalized collision energy (NCE) of 27.f

#### Data processing

The MS/MS spectra were searched against the UniProt (www.uniprot.org) *Canis lupus familiaris* database with common lab contaminants using Sequest within Proteome Discoverer 1.4. Searches were performed using the digestion enzyme trypsin permitting two missed cleavages, peptide length of 6 to 144, a precursor mass tolerance of ±10 ppm, a fragment mass tolerance of ±0.4 Da, variable modification of oxidation on methionine, deamidation on glutamine and asparagine, and a fixed modification of carbamidomethyl on cysteine. The results were filtered to better than ≤1% peptide; the protein false discovery rate (FDR) was determined by searching against a decoy database, and only proteins with at least two unique peptides were reported.

### Data presentation and statistical analysis

Unless otherwise indicated data points represent independent experiments. Explanation of their acquisition and number of independent experiments are noted in the figure legends. All analysis was carried out using GraphPad Prism. Error bars are represented as mean±s.d.. For comparison of two sample populations, we used a parametric two-tailed *t*-test (paired or unpaired as indicated in the figure legend) when sample values in individual experiments were normally distributed. Where normality could not be established, samples were compared by a nonparametric Mann–Whitney test. We used the convention for representation of *P*-values as follows: *P<0.05; **P<0.01, ***P<0.001.

## Supplementary Material



10.1242/joces.261705_sup1 Supplementary information

Table S1. List of proteins co-isolated with Borg5-HALO from MDCK lysates Sequence coverage and Average #PSM from 3 experiments for non-transfected control (C) and Borg5-HALO (W) transfected cell samples.

## References

[JCS261705C1] Adams, C. L., Chen, Y.-T., Smith, S. J. and Nelson, W. J. (1998). Mechanisms of epithelial cell-cell adhesion and cell compaction revealed by high-resolution tracking of E-cadherin-green fluorescent protein. *J. Cell Biol.* 142, 1105-1119. 10.1083/jcb.142.4.1105 9722621 PMC2132880

[JCS261705C2] Ageta-Ishihara, N., Miyata, T., Ohshima, C., Watanabe, M., Sato, Y., Hamamura, Y., Higashiyama, T., Mazitschek, R., Bito, H. and Kinoshita, M. (2013). Septins promote dendrite and axon development by negatively regulating microtubule stability via HDAC6-mediated deacetylation. *Nat. Commun.* 4, 2532. 10.1038/ncomms3532 24113571 PMC3826633

[JCS261705C3] Bai, X., Bowen, J. R., Knox, T. K., Zhou, K., Pendziwiat, M., Kuhlenbäumer, G., Sindelar, C. V. and Spiliotis, E. T. (2013). Novel septin 9 repeat motifs altered in neuralgic amyotrophy bind and bundle microtubules. *J. Cell Biol.* 203, 895-905. 10.1083/jcb.201308068 24344182 PMC3871440

[JCS261705C4] Benoit, B., Poüs, C. and Baillet, A. (2023). Septins as membrane influencers: direct play or in association with other cytoskeleton partners. *Front. Cell Dev. Biol.* 11, 1112319. 10.3389/fcell.2023.1112319 36875762 PMC9982393

[JCS261705C5] Blanchard, G. B., Murugesu, S., Adams, R. J., Martinez-Arias, A. and Gorfinkiel, N. (2010). Cytoskeletal dynamics and supracellular organisation of cell shape fluctuations during dorsal closure. *Development* 137, 2743-2752. 10.1242/dev.045872 20663818

[JCS261705C6] Borghi, N., Sorokina, M., Shcherbakova, O. G., Weis, W. I., Pruitt, B. L., Nelson, W. J. and Dunn, A. R. (2012). E-cadherin is under constitutive actomyosin-generated tension that is increased at cell-cell contacts upon externally applied stretch. *Proc. Natl. Acad. Sci. USA* 109, 12568-12573. 10.1073/pnas.1204390109 22802638 PMC3411997

[JCS261705C7] Bowen, J. R., Hwang, D., Bai, X., Roy, D. and Spiliotis, E. T. (2011). Septin GTPases spatially guide microtubule organization and plus end dynamics in polarizing epithelia. *J. Cell Biol.* 194, 187-197. 10.1083/jcb.201102076 21788367 PMC3144415

[JCS261705C8] Bridges, A. A., Zhang, H., Mehta, S. B., Occhipinti, P., Tani, T. and Gladfelter, A. S. (2014). Septin assemblies form by diffusion-driven annealing on membranes. *Proc. Natl. Acad. Sci. USA* 111, 2146-2151. 10.1073/pnas.1314138111 24469790 PMC3926015

[JCS261705C9] Brückner, B. R. and Janshoff, A. (2018). Importance of integrity of cell-cell junctions for the mechanics of confluent MDCK II cells. *Sci. Rep.* 8, 14117. 10.1038/s41598-018-32421-2 30237412 PMC6148251

[JCS261705C10] Burbelo, P. D., Snow, D. M., Bahou, W. and Spiegel, S. (1999). MSE55, a Cdc42 effector protein, induces long cellular extensions in fibroblasts. *Proc. Natl. Acad. Sci. USA* 96, 9083-9088. 10.1073/pnas.96.16.9083 10430899 PMC17736

[JCS261705C11] Calvo, F., Ranftl, R., Hooper, S., Farrugia, A. J., Moeendarbary, E., Bruckbauer, A., Batista, F., Charras, G. and Sahai, E. (2015). Cdc42EP3/BORG2 and septin network enables mechano-transduction and the emergence of cancer-associated fibroblasts. *Cell Rep.* 13, 2699-2714. 10.1016/j.celrep.2015.11.052 26711338 PMC4700053

[JCS261705C12] Campbell, B. R., Chen, Z., Faden, D. L., Agrawal, N., Li, R. J., Hanna, G. J., Iyer, N. G., Boot, A., Rozen, S. G., Vettore, A. L. et al. (2021). The mutational landscape of early- and typical-onset oral tongue squamous cell carcinoma. *Cancer* 127, 544-553. 10.1002/cncr.33309 33146897 PMC7891879

[JCS261705C13] Castro, D. K. S. V., Rosa, H. V. D., Mendonca, D. C., Cavini, I. A., Araujo, A. P. U. and Garratt, R. C. (2023). Dissecting the binding interface of the septin polymerization enhancer Borg BD3. *J. Mol. Biol.* 435, 168132. 10.1016/j.jmb.2023.168132 37121395

[JCS261705C14] Castro-Linares, G., Den Haan, J., Iv, F., Silva Martins, C., Bertin, A., Mavrakis, M. and Koenderink, G. H. (2022). Purification and quality control of recombinant septin complexes for cell-free reconstitution. *J. Vis. Exp.* 184, 63871. 10.3791/63871 35815970

[JCS261705C15] Cavey, M., Rauzi, M., Lenne, P.-F. and Lecuit, T. (2008). A two-tiered mechanism for stabilization and immobilization of E-cadherin. *Nature* 453, 751-756. 10.1038/nature06953 18480755

[JCS261705C16] Citi, S. (2019). The mechanobiology of tight junctions. *Biophys. Rev.* 11, 783-793. 10.1007/s12551-019-00582-7 31586306 PMC6815314

[JCS261705C17] Cohen, S., Kovari, D. T., Wei, W., Keate, R., Curtis, J. E. and Nie, S. (2018). Cdc42 regulates the cellular localization of Cdc42ep1 in controlling neural crest cell migration. *J. Mol. Cell. Biol.* 10, 376-387. 10.1093/jmcb/mjx044 29040749 PMC6692865

[JCS261705C18] Dolat, L. and Spiliotis, E. T. (2016). Septins promote macropinosome maturation and traffic to the lysosome by facilitating membrane fusion. *J. Cell Biol.* 214, 517-527. 10.1083/jcb.201603030 27551056 PMC5004444

[JCS261705C19] Dolat, L., Hunyara, J. L., Bowen, J. R., Karasmanis, E. P., Elgawly, M., Galkin, V. E. and Spiliotis, E. T. (2014). Septins promote stress fiber-mediated maturation of focal adhesions and renal epithelial motility. *J. Cell Biol.* 207, 225-235. 10.1083/jcb.201405050 25349260 PMC4210437

[JCS261705C20] Elbert, M., Cohen, D. and Müsch, A. (2006). PAR1b promotes cell-cell adhesion and inhibits dishevelled-mediated transformation of Madin-Darby canine kidney cells. *Mol. Biol. Cell* 17, 3345-3355. 10.1091/mbc.e06-03-0193 16707567 PMC1525229

[JCS261705C21] Even-Ram, S., Doyle, A. D., Conti, M. A., Matsumoto, K., Adelstein, R. S. and Yamada, K. M. (2007). Myosin IIA regulates cell motility and actomyosin-microtubule crosstalk. *Nat. Cell Biol.* 9, 299-309. 10.1038/ncb1540 17310241

[JCS261705C22] Farrugia, A. J. and Calvo, F. (2016). The Borg family of Cdc42 effector proteins Cdc42EP1-5. *Biochem. Soc. Trans.* 44, 1709-1716. 10.1042/BST20160219 27913681 PMC5134998

[JCS261705C23] Farrugia, A. J. and Calvo, F. (2017). Cdc42 regulates Cdc42EP3 function in cancer-associated fibroblasts. *Small GTPases* 8, 49-57. 10.1080/21541248.2016.1194952 27248291 PMC5331892

[JCS261705C24] Farrugia, A. J., Rodríguez, J., Orgaz, J. L., Lucas, M., Sanz-Moreno, V. and Calvo, F. (2020). CDC42EP5/BORG3 modulates SEPT9 to promote actomyosin function, migration, and invasion. *J. Cell Biol.* 219, e201912159. 10.1083/jcb.201912159 32798219 PMC7480113

[JCS261705C25] Field, C. M., Al-Awar, O., Rosenblatt, J., Wong, M. L., Alberts, B. and Mitchison, T. J. (1996). A purified Drosophila septin complex forms filaments and exhibits GTPase activity. *J. Cell Biol.* 133, 605-616. 10.1083/jcb.133.3.605 8636235 PMC2120824

[JCS261705C26] Foty, R. (2011). A simple hanging drop cell culture protocol for generation of 3D spheroids. *J. Vis. Exp.* 51, 2720. 10.3791/2720-v PMC3197119 21587162

[JCS261705C27] Hagiwara, A., Tanaka, Y., Hikawa, R., Morone, N., Kusumi, A., Kimura, H. and Kinoshita, M. (2011). Submembranous septins as relatively stable components of actin-based membrane skeleton. *Cytoskeleton. (Hoboken)* 68, 512-525. 10.1002/cm.20528 21800439

[JCS261705C28] Harwood, M. D., Zettl, K., Weinheimer, M., Pilla-Reddy, V., Shen, H., Jacobs, F., Chu, X., Huth, F., Nakakariya, M., Chothe, P. P. et al. (2023). Interlaboratory variability in the Madin-Darby canine kidney cell proteome. *Mol. Pharm.* 20, 3505-3518. 10.1021/acs.molpharmaceut.3c00108 37283406 PMC10324398

[JCS261705C29] Hatte, G., Prigent, C. and Tassan, J. P. (2018). Tight junctions negatively regulate mechanical forces applied to adherens junctions in vertebrate epithelial tissue. *J. Cell Sci.* 131, jcs208736. 10.1242/jcs.208736 29246943

[JCS261705C30] Hirsch, D. S., Pirone, D. M. and Burbelo, P. D. (2001). A new family of Cdc42 effector proteins, CEPs, function in fibroblast and epithelial cell shape changes. *J. Biol. Chem.* 276, 875-883. 10.1074/jbc.M007039200 11035016

[JCS261705C31] Ishizaki, T., Naito, M., Fujisawa, K., Maekawa, M., Watanabe, N., Saito, Y. and Narumiya, S. (1997). p160ROCK, a Rho-associated coiled-coil forming protein kinase, works downstream of Rho and induces focal adhesions. *FEBS Lett.* 404, 118-124. 10.1016/S0014-5793(97)00107-5 9119047

[JCS261705C32] Iv, F., Martins, C. S., Castro-Linares, G., Taveneau, C., Barbier, P., Verdier-Pinard, P., Camoin, L., Audebert, S., Tsai, F.-C., Ramond, L. et al. (2021). Insights into animal septins using recombinant human septin octamers with distinct SEPT9 isoforms. *J. Cell Sci.* 134, jcs258484. 10.1242/jcs.258484 34350965

[JCS261705C33] Joberty, G., Perlungher, R. R. and Macara, I. G. (1999). The Borgs, a new family of Cdc42 and TC10 GTPase-interacting proteins. *Mol. Cell. Biol.* 19, 6585-6597. 10.1128/MCB.19.10.6585 10490598 PMC84628

[JCS261705C34] Joberty, G., Perlungher, R. R., Sheffield, P. J., Kinoshita, M., Noda, M., Haystead, T. and Macara, I. G. (2001). Borg proteins control septin organization and are negatively regulated by Cdc42. *Nat. Cell Biol.* 3, 861-866. 10.1038/ncb1001-861 11584266

[JCS261705C35] Joo, E., Surka, M. C. and Trimble, W. S. (2007). Mammalian SEPT2 is required for scaffolding nonmuscle myosin II and its kinases. *Dev. Cell* 13, 677-690. 10.1016/j.devcel.2007.09.001 17981136

[JCS261705C36] Kalaji, R., Wheeler, A. P., Erasmus, J. C., Lee, S. Y., Endres, R. G., Cramer, L. P. and Braga, V. M. M. (2012). ROCK1 and ROCK2 regulate epithelial polarisation and geometric cell shape. *Biol. Cell* 104, 435-451. 10.1111/boc.201100093 22462535

[JCS261705C37] Kaverina, I., Rottner, K. and Small, J. V. (1998). Targeting, capture, and stabilization of microtubules at early focal adhesions. *J. Cell Biol.* 142, 181-190. 10.1083/jcb.142.1.181 9660872 PMC2133026

[JCS261705C38] Kinoshita, M. (2003). Assembly of mammalian septins. *J. Biochem.* 134, 491-496. 10.1093/jb/mvg182 14607974

[JCS261705C39] Kinoshita, M., Kumar, S., Mizoguchi, A., Ide, C., Kinoshita, A., Haraguchi, T., Hiraoka, Y. and Noda, M. (1997). Nedd5, a mammalian septin, is a novel cytoskeletal component interacting with actin-based structures. *Genes Dev.* 11, 1535-1547. 10.1101/gad.11.12.1535 9203580

[JCS261705C40] Kinoshita, M., Field, C. M., Coughlin, M. L., Straight, A. F. and Mitchison, T. J. (2002). Self- and actin-templated assembly of Mammalian septins. *Dev. Cell* 3, 791-802. 10.1016/S1534-5807(02)00366-0 12479805

[JCS261705C41] Klingner, C., Cherian, A. V., Fels, J., Diesinger, P. M., Aufschnaiter, R., Maghelli, N., Keil, T., Beck, G., Tolić-Nørrelykke, I. M., Bathe, M. et al. (2014). Isotropic actomyosin dynamics promote organization of the apical cell cortex in epithelial cells. *J. Cell Biol.* 207, 107-121. 10.1083/jcb.201402037 25313407 PMC4195824

[JCS261705C42] Köster, D. V., Husain, K., Iljazi, E., Bhat, A., Bieling, P., Mullins, R. D., Rao, M. and Mayor, S. (2016). Actomyosin dynamics drive local membrane component organization in an in vitro active composite layer. *Proc. Natl. Acad. Sci. USA* 113, E1645-E1654. 10.1073/pnas.1514030113 26929326 PMC4812753

[JCS261705C43] Kremer, B. E., Haystead, T. and Macara, I. G. (2005). Mammalian septins regulate microtubule stability through interaction with the microtubule-binding protein MAP4. *Mol. Biol. Cell* 16, 4648-4659. 10.1091/mbc.e05-03-0267 16093351 PMC1237071

[JCS261705C44] Kuzmić, M., Castro Linares, G., Leischner Fialová, J., Iv, F., Salaün, D., Llewellyn, A., Gomes, M., Belhabib, M., Liu, Y., Asano, K. et al. (2022). Septin-microtubule association via a motif unique to isoform 1 of septin 9 tunes stress fibers. *J. Cell Sci.* 135, jcs258850. 10.1242/jcs.258850 34854883

[JCS261705C45] Lam, M. and Calvo, F. (2019). Regulation of mechanotransduction: Emerging roles for septins. *Cytoskeleton (Hoboken)* 76, 115-122. 10.1002/cm.21485 30091182 PMC6519387

[JCS261705C46] Lamouille, S., Xu, J. and Derynck, R. (2014). Molecular mechanisms of epithelial-mesenchymal transition. *Nat. Rev. Mol. Cell Biol.* 15, 178-196. 10.1038/nrm3758 24556840 PMC4240281

[JCS261705C47] Lehtimäki, J. I., Rajakylä, E. K., Tojkander, S. and Lappalainen, P. (2021). Generation of stress fibers through myosin-driven reorganization of the actin cortex. *eLife* 10, e60710. 10.7554/eLife.60710 33506761 PMC7877910

[JCS261705C48] Liu, Z., Vong, Q. P., Liu, C. and Zheng, Y. (2014). Borg5 is required for angiogenesis by regulating persistent directional migration of the cardiac microvascular endothelial cells. *Mol. Biol. Cell* 25, 841-851. 10.1091/mbc.e13-09-0543 24451259 PMC3952853

[JCS261705C49] Loza, A. J., Koride, S., Schimizzi, G. V., Li, B., Sun, S. X. and Longmore, G. D. (2016). Cell density and actomyosin contractility control the organization of migrating collectives within an epithelium. *Mol. Biol. Cell* 27, 3459-3470. 10.1091/mbc.e16-05-0329 27605707 PMC5221580

[JCS261705C50] Lynn, K. S., Peterson, R. J. and Koval, M. (2020). Ruffles and spikes: control of tight junction morphology and permeability by claudins. *Biochim. Biophys. Acta Biomembr.* 1862, 183339. 10.1016/j.bbamem.2020.183339 32389670 PMC7299829

[JCS261705C51] Martin, A. C. (2020). The physical mechanisms of drosophila gastrulation: mesoderm and endoderm invagination. *Genetics* 214, 543-560. 10.1534/genetics.119.301292 32132154 PMC7054018

[JCS261705C52] Martínez-Ara, G., Taberner, N., Takayama, M., Sandaltzopoulou, E., Villava, C. E., Bosch-Padrós, M., Takata, N., Trepat, X., Eiraku, M. and Ebisuya, M. (2022). Optogenetic control of apical constriction induces synthetic morphogenesis in mammalian tissues. *Nat. Commun.* 13, 5400. 10.1038/s41467-022-33115-0 36104355 PMC9474505

[JCS261705C53] Martins, C. S., Taveneau, C., Castro-Linares, G., Baibakov, M., Buzhinsky, N., Eroles, M., Milanović, V., Omi, S., Pedelacq, J. D., Iv, F. et al. (2023). Human septins organize as octamer-based filaments and mediate actin-membrane anchoring in cells. *J. Cell Biol.* 222, e202203016. 10.1083/jcb.202203016 36562751 PMC9802686

[JCS261705C54] Maruthamuthu, V., Sabass, B., Schwarz, U. S. and Gardel, M. L. (2011). Cell-ECM traction force modulates endogenous tension at cell-cell contacts. *Proc. Natl. Acad. Sci. USA* 108, 4708-4713. 10.1073/pnas.1011123108 21383129 PMC3064395

[JCS261705C55] Mavrakis, M., Azou-Gros, Y., Tsai, F.-C., Alvarado, J., Bertin, A., Iv, F., Kress, A., Brasselet, S., Koenderink, G. H. and Lecuit, T. (2014). Septins promote F-actin ring formation by crosslinking actin filaments into curved bundles. *Nat. Cell Biol.* 16, 322-334. 10.1038/ncb2921 24633326

[JCS261705C56] Miller, C. J., Harris, D., Weaver, R., Ermentrout, G. B. and Davidson, L. A. (2018). Emergent mechanics of actomyosin drive punctuated contractions and shape network morphology in the cell cortex. *PLoS Comput. Biol.* 14, e1006344. 10.1371/journal.pcbi.1006344 30222728 PMC6171965

[JCS261705C57] Morris, H. T. and Machesky, L. M. (2015). Actin cytoskeletal control during epithelial to mesenchymal transition: focus on the pancreas and intestinal tract. *Br. J. Cancer* 112, 613-620. 10.1038/bjc.2014.658 25611303 PMC4333498

[JCS261705C58] Munro, E., Nance, J. and Priess, J. R. (2004). Cortical flows powered by asymmetrical contraction transport PAR proteins to establish and maintain anterior-posterior polarity in the early C. elegans embryo. *Dev. Cell* 7, 413-424. 10.1016/j.devcel.2004.08.001 15363415

[JCS261705C59] Nakahara, S., Tsutsumi, K., Zuinen, T. and Ohta, Y. (2015). FilGAP, a Rho-ROCK-regulated GAP for Rac, controls adherens junctions in MDCK cells. *J. Cell Sci.* 128, 2047-2056. 10.1242/jcs.160192 25908853

[JCS261705C60] Nakano, K., Takaishi, K., Kodama, A., Mammoto, A., Shiozaki, H., Monden, M. and Takai, Y. (1999). Distinct actions and cooperative roles of ROCK and mDia in Rho small G protein-induced reorganization of the actin cytoskeleton in Madin-Darby canine kidney cells. *Mol. Biol. Cell* 10, 2481-2491. 10.1091/mbc.10.8.2481 10436006 PMC25478

[JCS261705C61] Nakos, K., Alam, M. N. A., Radler, M. R., Kesisova, I. A., Yang, C., Okletey, J., Tomasso, M. R., Padrick, S. B., Svitkina, T. M. and Spiliotis, E. T. (2022). Septins mediate a microtubule-actin crosstalk that enables actin growth on microtubules. *Proc. Natl. Acad. Sci. USA* 119, e2202803119. 10.1073/pnas.2202803119 36475946 PMC9897426

[JCS261705C62] Nelson, W. J. (2009). Remodeling epithelial cell organization: transitions between front-rear and apical-basal polarity. *Cold Spring Harb. Perspect. Biol.* 1, a000513. 10.1101/cshperspect.a000513 20066074 PMC2742086

[JCS261705C63] Odenwald, M. A., Choi, W., Kuo, W. T., Singh, G., Sailer, A., Wang, Y., Shen, L., Fanning, A. S. and Turner, J. R. (2018). The scaffolding protein ZO-1 coordinates actomyosin and epithelial apical specializations in vitro and in vivo. *J. Biol. Chem.* 293, 17317-17335. 10.1074/jbc.RA118.003908 30242130 PMC6231134

[JCS261705C64] Rafiq, N. B. M., Nishimura, Y., Plotnikov, S. V., Thiagarajan, V., Zhang, Z., Shi, S., Natarajan, M., Viasnoff, V., Kanchanawong, P., Jones, G. E. et al. (2019). A mechano-signalling network linking microtubules, myosin IIA filaments and integrin-based adhesions. *Nat. Mater.* 18, 638-649. 10.1038/s41563-019-0371-y 31114072

[JCS261705C65] Rajakylä, E. K., Lehtimäki, J. I., Acheva, A., Schaible, N., Lappalainen, P., Krishnan, R. and Tojkander, S. (2020). Assembly of peripheral actomyosin bundles in epithelial cells is dependent on the CaMKK2/AMPK pathway. *Cell Rep.* 30, 4266-4280.e4. 10.1016/j.celrep.2020.02.096 32209483

[JCS261705C66] Rouaud, F., Huang, W., Flinois, A., Jain, K., Vasileva, E., Di Mattia, T., Mauperin, M., Parry, D. A. D., Dugina, V., Chaponnier, C. et al. (2023). Cingulin and paracingulin tether myosins-2 to junctions to mechanoregulate the plasma membrane. *J. Cell Biol.* 222, e202208065. 10.1083/jcb.202208065 37204781 PMC10202830

[JCS261705C67] Sadati, M., Taheri Qazvini, N., Krishnan, R., Park, C. Y. and Fredberg, J. J. (2013). Collective migration and cell jamming. *Differentiation* 86, 121-125. 10.1016/j.diff.2013.02.005 23791490 PMC3795803

[JCS261705C68] Salameh, J., Cantaloube, I., Benoit, B., Poüs, C. and Baillet, A. (2021). Cdc42 and its BORG2 and BORG3 effectors control the subcellular localization of septins between actin stress fibers and microtubules. *Curr. Biol.* 31, 4088-4103.e5. 10.1016/j.cub.2021.07.004 34329591

[JCS261705C69] Sato, Y., Kamijo, K., Tsutsumi, M., Murakami, Y. and Takahashi, M. (2020). Nonmuscle myosin IIA and IIB differently suppress microtubule growth to stabilize cell morphology. *J. Biochem.* 167, 25-39. 10.1093/jb/mvz082 31599953

[JCS261705C70] Scarpa, E., Szabó, A., Bibonne, A., Theveneau, E., Parsons, M. and Mayor, R. (2015). Cadherin switch during EMT in neural crest cells leads to contact inhibition of locomotion via repolarization of forces. *Dev. Cell* 34, 421-434. 10.1016/j.devcel.2015.06.012 26235046 PMC4552721

[JCS261705C71] Schmidt, K. and Nichols, B. J. (2004). Functional interdependence between septin and actin cytoskeleton. *BMC Cell Biol.* 5, 43. 10.1186/1471-2121-5-43 15541171 PMC535351

[JCS261705C72] Seetharaman, S. and Etienne-Manneville, S. (2019). Microtubules at focal adhesions - a double-edged sword. *J. Cell Sci.* 132, jcs232843. 10.1242/jcs.232843 31597743

[JCS261705C73] Sellin, M. E., Sandblad, L., Stenmark, S. and Gullberg, M. (2011). Deciphering the rules governing assembly order of mammalian septin complexes. *Mol. Biol. Cell* 22, 3152-3164. 10.1091/mbc.e11-03-0253 21737677 PMC3164462

[JCS261705C74] Sellin, M. E., Stenmark, S. and Gullberg, M. (2014). Cell type-specific expression of SEPT3-homology subgroup members controls the subunit number of heteromeric septin complexes. *Mol. Biol. Cell* 25, 1594-1607. 10.1091/mbc.e13-09-0553 24648497 PMC4019491

[JCS261705C75] Shao, H., Wu, C. and Wells, A. (2010). Phosphorylation of alpha-actinin 4 upon epidermal growth factor exposure regulates its interaction with actin. *J. Biol. Chem.* 285, 2591-2600. 10.1074/jbc.M109.035790 19920151 PMC2807316

[JCS261705C76] Sheffield, P. J., Oliver, C. J., Kremer, B. E., Sheng, S., Shao, Z. and Macara, I. G. (2003). Borg/septin interactions and the assembly of mammalian septin heterodimers, trimers, and filaments. *J. Biol. Chem.* 278, 3483-3488. 10.1074/jbc.M209701200 12446710

[JCS261705C77] Shin, K.-J., Wall, E. A., Zavzavadjian, J. R., Santat, L. A., Liu, J., Hwang, J.-I., Rebres, R., Roach, T., Seaman, W., Simon, M. I. et al. (2006). A single lentiviral vector platform for microRNA-based conditional RNA interference and coordinated transgene expression. *Proc. Natl. Acad. Sci. USA* 103, 13759-13764. 10.1073/pnas.0606179103 16945906 PMC1557799

[JCS261705C78] Sidhaye, V. K., Chau, E., Breysse, P. N. and King, L. S. (2011). Septin-2 mediates airway epithelial barrier function in physiologic and pathologic conditions. *Am. J. Respir. Cell Mol. Biol.* 45, 120-126. 10.1165/rcmb.2010-0235OC 20870893 PMC3145065

[JCS261705C79] Sirajuddin, M., Farkasovsky, M., Hauer, F., Kühlmann, D., Macara, I. G., Weyand, M., Stark, H. and Wittinghofer, A. (2007). Structural insight into filament formation by mammalian septins. *Nature* 449, 311-315. 10.1038/nature06052 17637674

[JCS261705C80] Smith, C., Dolat, L., Angelis, D., Forgacs, E., Spiliotis, E. T. and Galkin, V. E. (2015). Septin 9 exhibits polymorphic binding to F-actin and inhibits myosin and cofilin activity. *J. Mol. Biol.* 427, 3273-3284. 10.1016/j.jmb.2015.07.026 26297986 PMC4587343

[JCS261705C81] Soroor, F., Kim, M. S., Palander, O., Balachandran, Y., Collins, R. F., Benlekbir, S., Rubinstein, J. L. and Trimble, W. S. (2021). Revised subunit order of mammalian septin complexes explains their in vitro polymerization properties. *Mol. Biol. Cell* 32, 289-300. 10.1091/mbc.E20-06-0398 33263440 PMC8098831

[JCS261705C82] Spiliotis, E. T. (2010). Regulation of microtubule organization and functions by septin GTPases. *Cytoskeleton* 67, 339-345. 10.1002/cm.20448 20517923

[JCS261705C83] Spiliotis, E. T. (2018). Spatial effects - site-specific regulation of actin and microtubule organization by septin GTPases. *J. Cell Sci.* 131, jcs207555. 10.1242/jcs.207555 29326311 PMC5818061

[JCS261705C84] Spiliotis, E. T. and Nakos, K. (2021). Cellular functions of actin- and microtubule-associated septins. *Curr. Biol.* 31, R651-R666. 10.1016/j.cub.2021.03.064 34033796 PMC8194058

[JCS261705C85] Spiliotis, E. T., Hunt, S. J., Hu, Q., Kinoshita, M. and Nelson, W. J. (2008). Epithelial polarity requires septin coupling of vesicle transport to polyglutamylated microtubules. *J. Cell Biol.* 180, 295-303. 10.1083/jcb.200710039 18209106 PMC2213583

[JCS261705C86] Stevenson, B. R., Siliciano, J. D., Mooseker, M. S. and Goodenough, D. A. (1986). Identification of ZO-1: a high molecular weight polypeptide associated with the tight junction (zonula occludens) in a variety of epithelia. *J. Cell Biol.* 103, 755-766. 10.1083/jcb.103.3.755 3528172 PMC2114282

[JCS261705C87] Tan, B., Yatim, S. M. J. M., Peng, S., Gunaratne, J., Hunziker, W. and Ludwig, A. (2020). The mammalian crumbs complex defines a distinct polarity domain apical of epithelial tight junctions. *Curr. Biol.* 30, 2791-2804.e6. 10.1016/j.cub.2020.05.032 32531288

[JCS261705C88] Targa, B., Klipfel, L., Cantaloube, I., Salameh, J., Benoit, B., Poüs, C. and Baillet, A. (2019). Septin filament coalignment with microtubules depends on SEPT9_i1 and tubulin polyglutamylation, and is an early feature of acquired cell resistance to paclitaxel. *Cell Death Dis.* 10, 54. 10.1038/s41419-019-1318-6 30670682 PMC6342940

[JCS261705C89] Thiery, J. P. (2002). Epithelial-mesenchymal transitions in tumour progression. *Nat. Rev. Cancer* 2, 442-454. 10.1038/nrc822 12189386

[JCS261705C90] Tokuda, S., Higashi, T. and Furuse, M. (2014). ZO-1 knockout by TALEN-mediated gene targeting in MDCK cells: involvement of ZO-1 in the regulation of cytoskeleton and cell shape. *PLoS ONE* 9, e104994. 10.1371/journal.pone.0104994 25157572 PMC4144852

[JCS261705C91] Tomasso, M. R. and Padrick, S. B. (2023). BORG family proteins in physiology and human disease. *Cytoskeleton* 80, 182-198. 10.1002/cm.21768 37403807

[JCS261705C92] Tse, J. R. and Engler, A. J. (2010). Preparation of hydrogel substrates with tunable mechanical properties. *Curr. Protoc. Cell Biol.* Chapter 10, Unit 10 16. 10.1002/0471143030.cb1016s47 20521229

[JCS261705C93] Vallenius, T. (2013). Actin stress fibre subtypes in mesenchymal-migrating cells. *Open Biol.* 3, 130001. 10.1098/rsob.130001 23782578 PMC3718327

[JCS261705C94] Vasquez, C. G., Tworoger, M. and Martin, A. C. (2014). Dynamic myosin phosphorylation regulates contractile pulses and tissue integrity during epithelial morphogenesis. *J. Cell Biol.* 206, 435-450. 10.1083/jcb.201402004 25092658 PMC4121972

[JCS261705C95] Verdier-Pinard, P., Salaun, D., Bouguenina, H., Shimada, S., Pophillat, M., Audebert, S., Agavnian, E., Coslet, S., Charafe-Jauffret, E., Tachibana, T. et al. (2017). Septin 9_i2 is downregulated in tumors, impairs cancer cell migration and alters subnuclear actin filaments. *Sci. Rep.* 7, 44976. 10.1038/srep44976 28338090 PMC5364497

[JCS261705C96] Vong, Q. P., Liu, Z., Yoo, J. G., Chen, R., Xie, W., Sharov, A. A., Fan, C.-M., Liu, C., Ko, M. S. H. and Zheng, Y. (2010). A role for borg5 during trophectoderm differentiation. *Stem Cells* 28, 1030-1038. 10.1002/stem.428 20506138 PMC2957878

[JCS261705C97] Wang, X., Wang, W., Wang, X., Wang, M., Zhu, L., Garba, F., Fu, C., Zieger, B., Liu, X., Liu, X. et al. (2021). The septin complex links the catenin complex to the actin cytoskeleton for establishing epithelial cell polarity. *J. Mol. Cell Biol.* 13, 395-408. 10.1093/jmcb/mjab036 34143183 PMC8436676

[JCS261705C98] Woods, B. L. and Gladfelter, A. S. (2021). The state of the septin cytoskeleton from assembly to function. *Curr. Opin. Cell Biol.* 68, 105-112. 10.1016/j.ceb.2020.10.007 33188984 PMC7952027

