# Supplementary material for: Borg5 restricts contractility and motility in epithelial MDCK cells
Source: J Cell Sci. 2024 Dec 10;137(23):jcs261705. doi: 10.1242/jcs.261705 (PMC11698036; doi:10.1242/jcs.261705)
Supplement: Supplementary information [file joces-137-261705-s1.pdf]

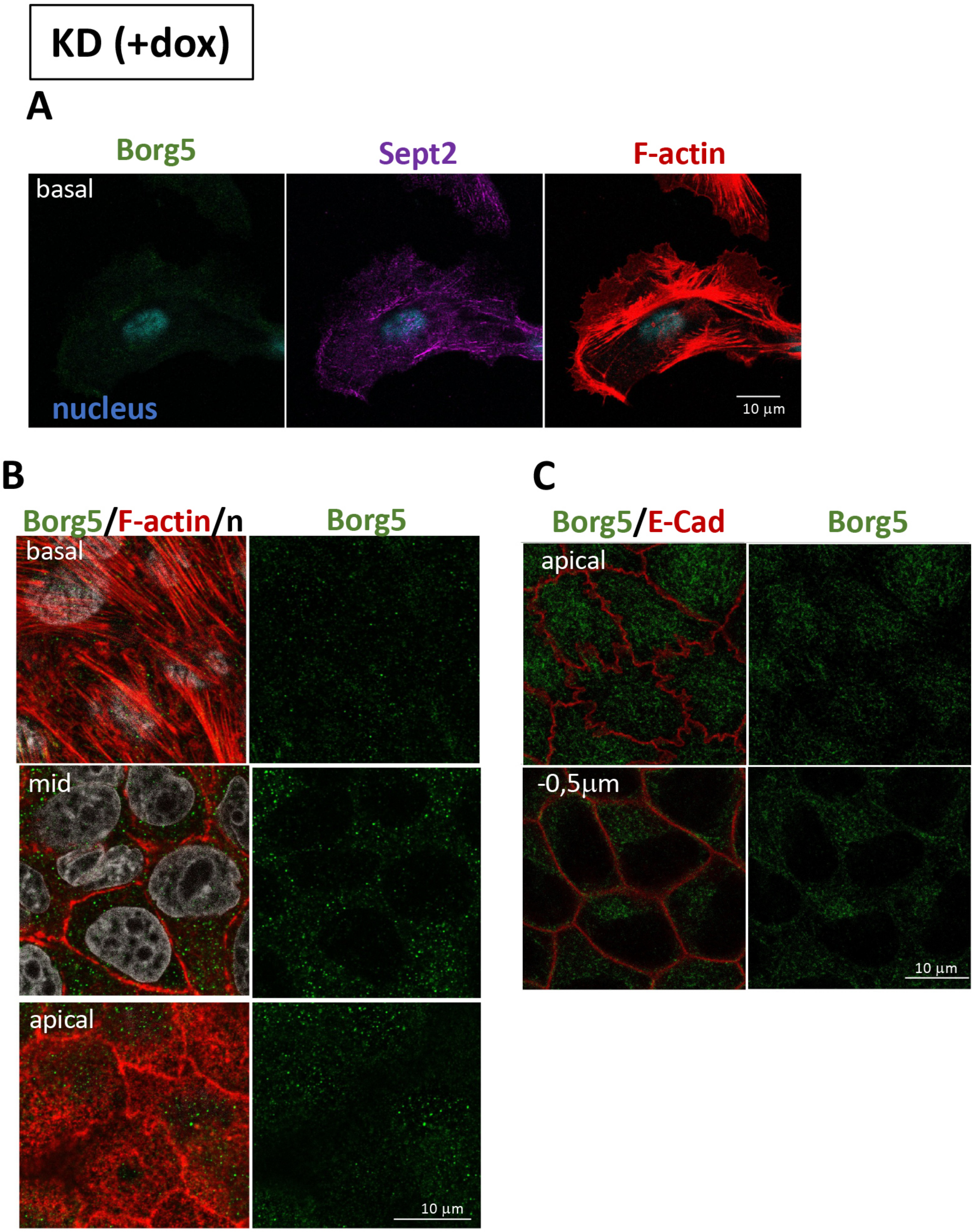

Fig. S1. Borg5 depletion abolishes the Borg5 IF signal, *related to Fig.1*

Borg5-depleted MDCK-Borg5 cells grown in dox (KD, +dox), corresponding to the uninduced control cells shown in Fig.1. **A)** single cells; **B,C)** confluent cells grown on glass (B) or on transwell inserts (C); Images are representative of 3 experiments.

Journal of Cell Science • Supplementary information

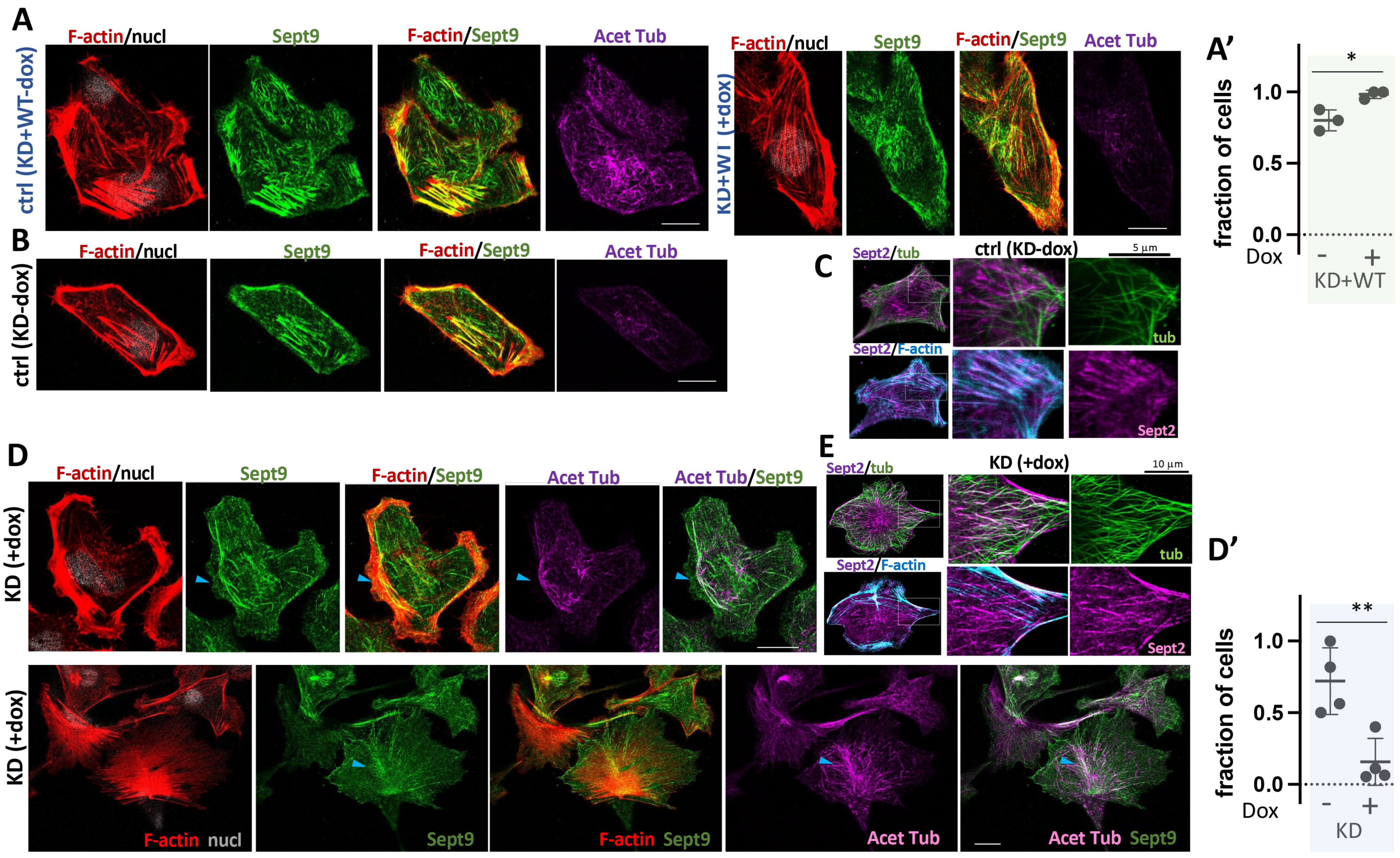

Fig. S2. Borg5 depletion decreases F-actin alignment of Septin 2 and 9, *related to Fig. 3*

A,B,C,D) Borg5 KD cells (KD) or Borg5KD+WT cells (KD+WT) cells in -/+dox were plated as single cells as in Fig.3 and co-labeled for either septin 9, F-actin and acetylated tubulin (Acet Tub)

(A,B,D) or for septin 2, F-actin and tubulin (C,E). Depicted are characteristic phenotypes for the respective cell lines. Blue arrowheads in D point to septin 9 alignment with Acet Tub rather than with F-actin; C,D: boxed areas in the cells (left panels) are amplified in the right panels. Scale bars: 10µm, unless noted otherwise.

A',D') fraction of cells with alignment of septin 9 and F-actin fiber at the attachment plane; 20 randomly imaged cells were scored; N=4 (Borg5KD), N=3 (Borg5KD+WT). **p=0.0074 *p=0.016 by unpaired two-tailed *t*-test.

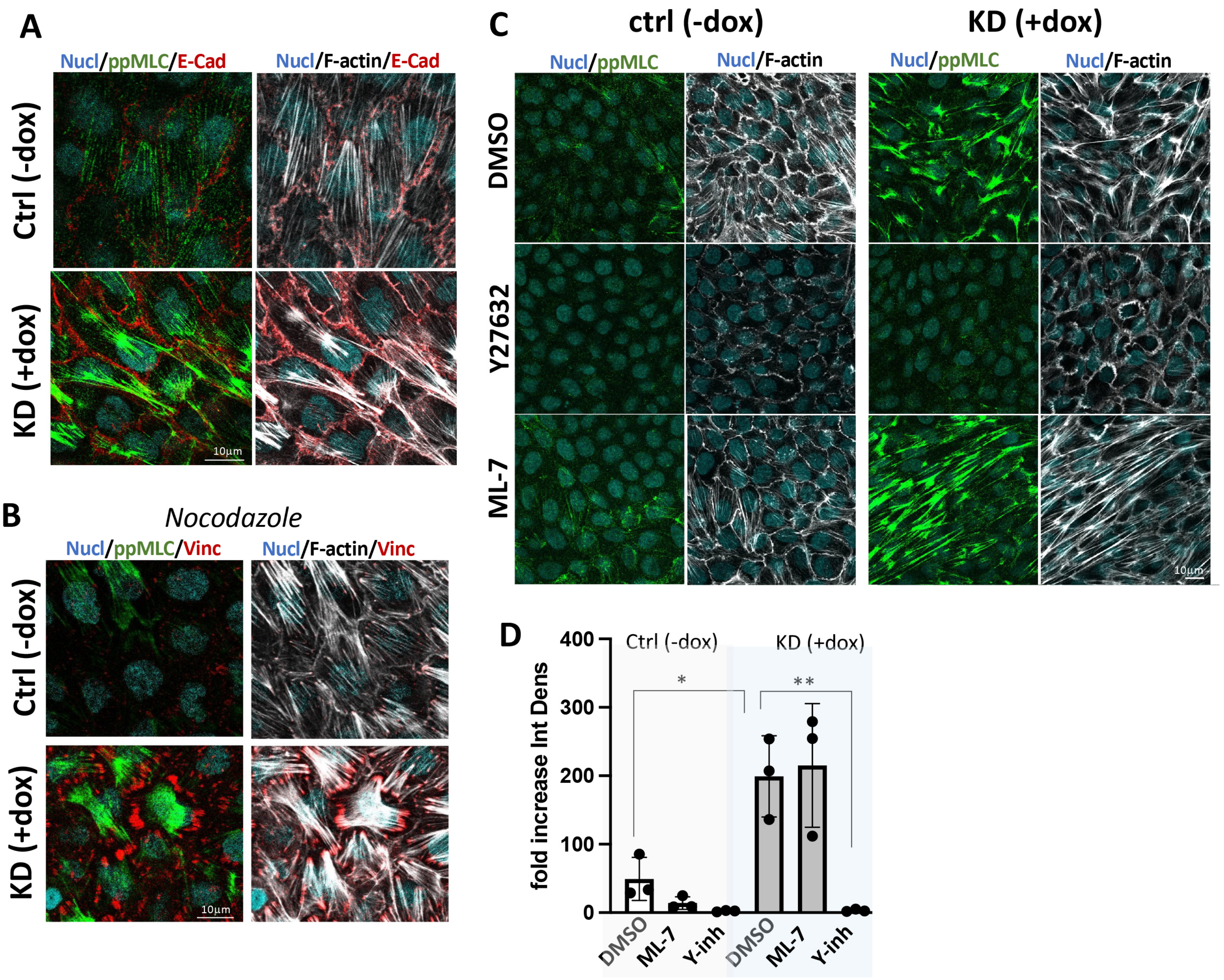

Fig. S3. Borg5 depletion increases Rho kinase (RhoK)-dependent myosin II activity in the center of stellate stress fibers, *related to Fig. 4*

A-D) Uninduced (ctrl,-dox) and induced Borg5 KD cells (KD,+dox) plated at $3x10^5$ cells/cm^2, were cultured for 24h, and treated for 2h with DMSO (A) or 10 µm nocodazole (B), or for 3h with DMSO or the Myosin Light Chain Kinase (MLCK) inhibitor ML-7 (30µM) or the RhoK inhibitor Y27632 (10 µM) (C) and analyzed for MyoII activity by IF for phosphorylated Myosin Light Chain 2 (ppMLC) and F-actin. Note that the stellate stress fibers in Borg-KD cells are highly enriched in and dependent on active MyoII, which is controlled by RhoK rather than MLCK; also note that ppMLC is concentrated in the center of stellate stress fibers;

D) Integrated fluorescence density of basal ppMLC from 3 random images such as those in C normalized as fold increase of the lowest measured density in a data set; N=3, *p=0.02, ** p=0.008 by paired two-tailed *t*-test.

Journal of Cell Science • Supplementary information

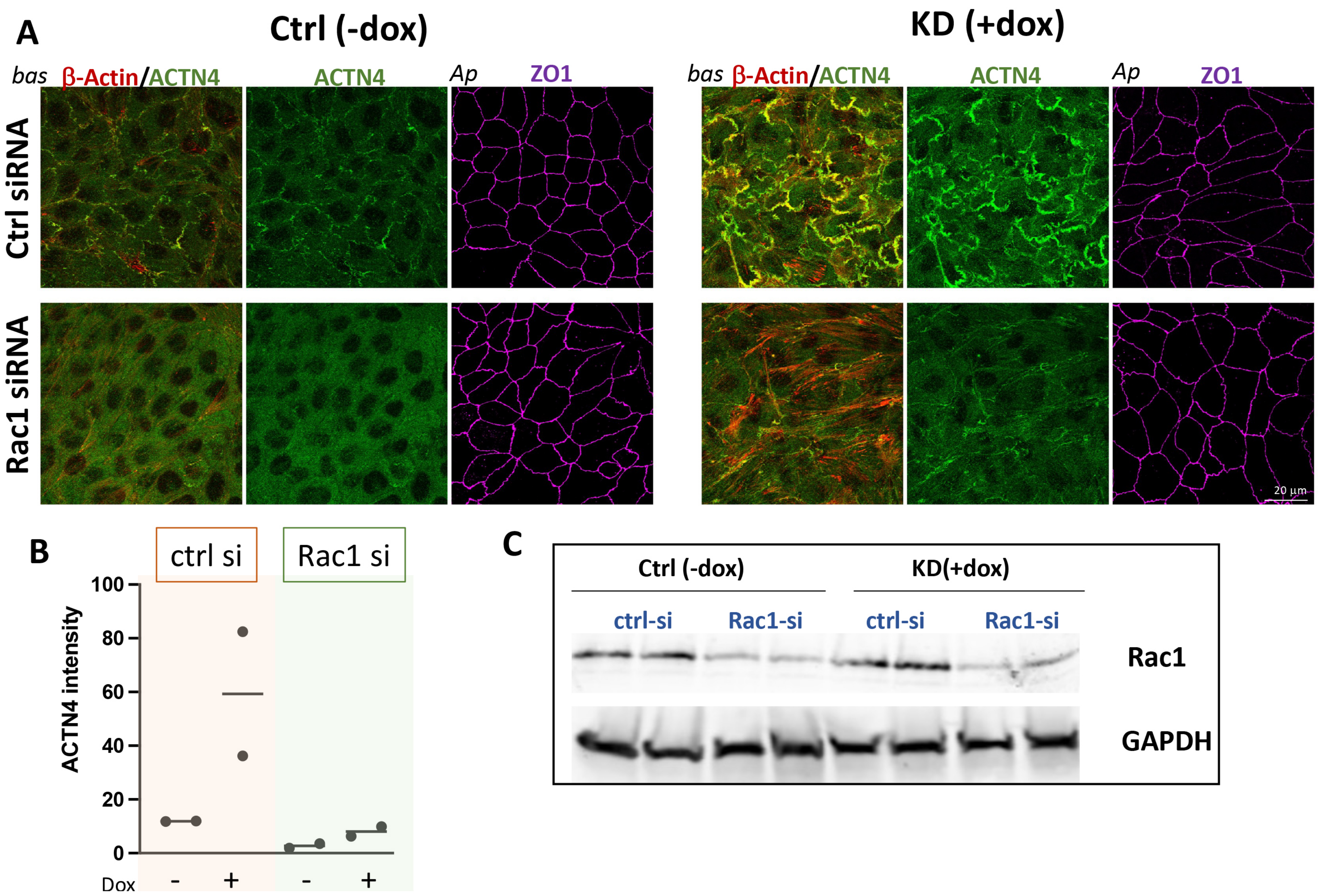

Fig. S4. Rac1 depletion inhibits Borg5-induced ACTN4-positive ruffles, *related to Fig. 4*

Rac1-depleted uninduced (ctrl,-dox) or induced (KD, +dox) Borg5 KD cells were plated at $3x10^5$ cells/cm^2 cultured for 24h and analyzed for ACTN4 and actin-positive ruffles **(A,B)**. Cells were also labeled for ZO1, indicating less cell stretching in Rac1-depleted KD+dox cells. **B)** Normalized ACTN4 intensity of 3 images from N=2 was plotted; C: Rac1 levels from duplicate lysates were analyzed by IB; GAPDH is shown for normalization. Blots are representative of 2 experiments.

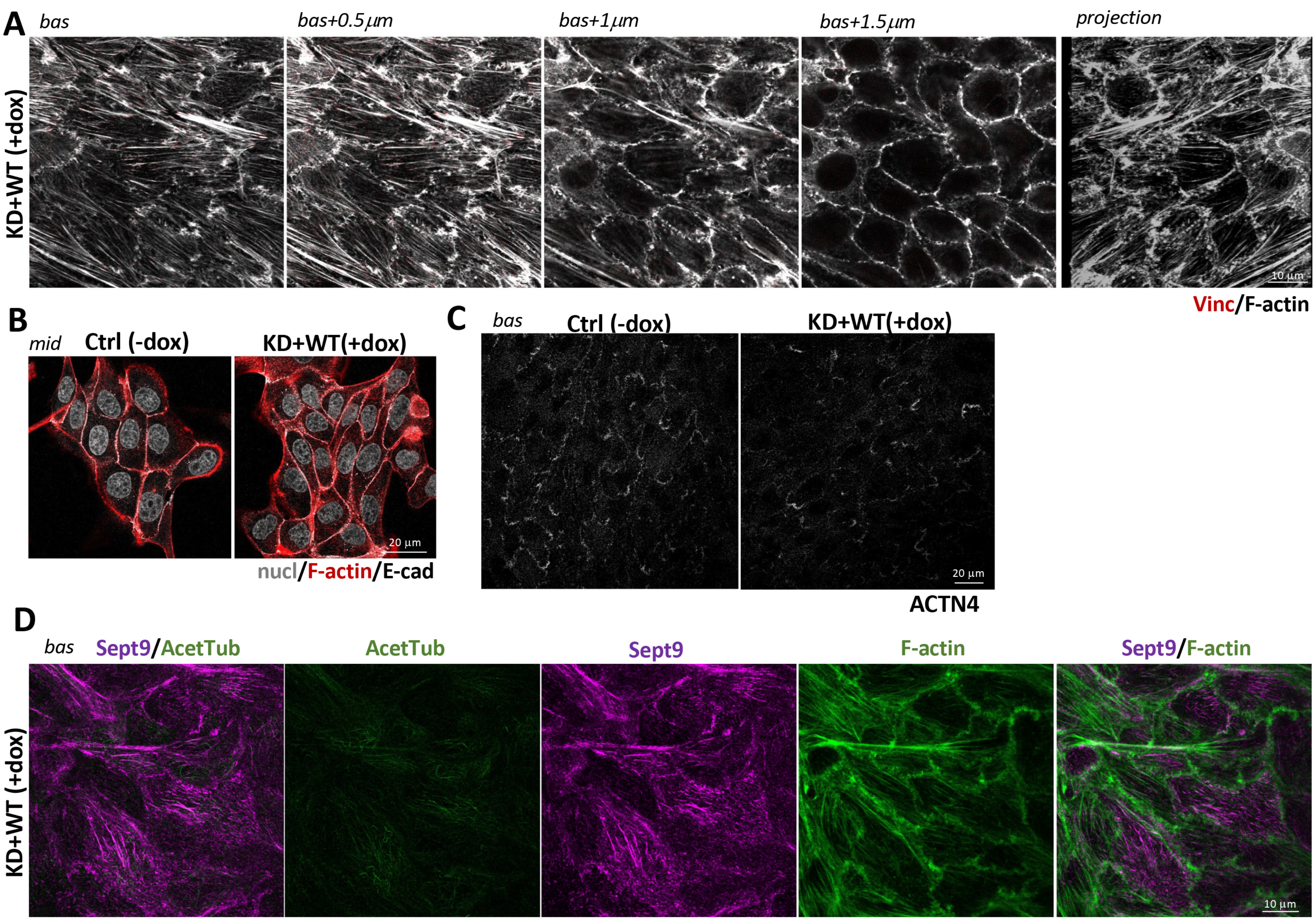

Fig. S5. expression of mouse Borg5WT in Borg5KD cells rescues the F-actin and acetylated MT-phenotypes, *related to Fig. 4*

Uninduced (ctrl,-dox) or induced (KD+WT, +dox) Borg5KD+WT cells plated at either 10^5 cells/cm^2 (**A,C,D**) or at $3x10^5$ cells/cm^2 (**B)** were cultured for 24h and processed for IF. Shown are confocal sections at the basal domain (bas), sections +0.5, +1 and 1.5 μm above the basal domain (A: bas +0.5 to 1.5) or cell mid-sections (mid) of IF labeled cells like the images in Fig.3. Note that WT expression prevents the formation of stellate stress fibers (A), reduced compaction (B), ACTN4 ruffles (C), and lacked the basal septin-aligned acetylated tubulin seen in KD cells (D). Quantitation of the phenotype rescue is in Fig. 3.

Journal of Cell Science • Supplementary information

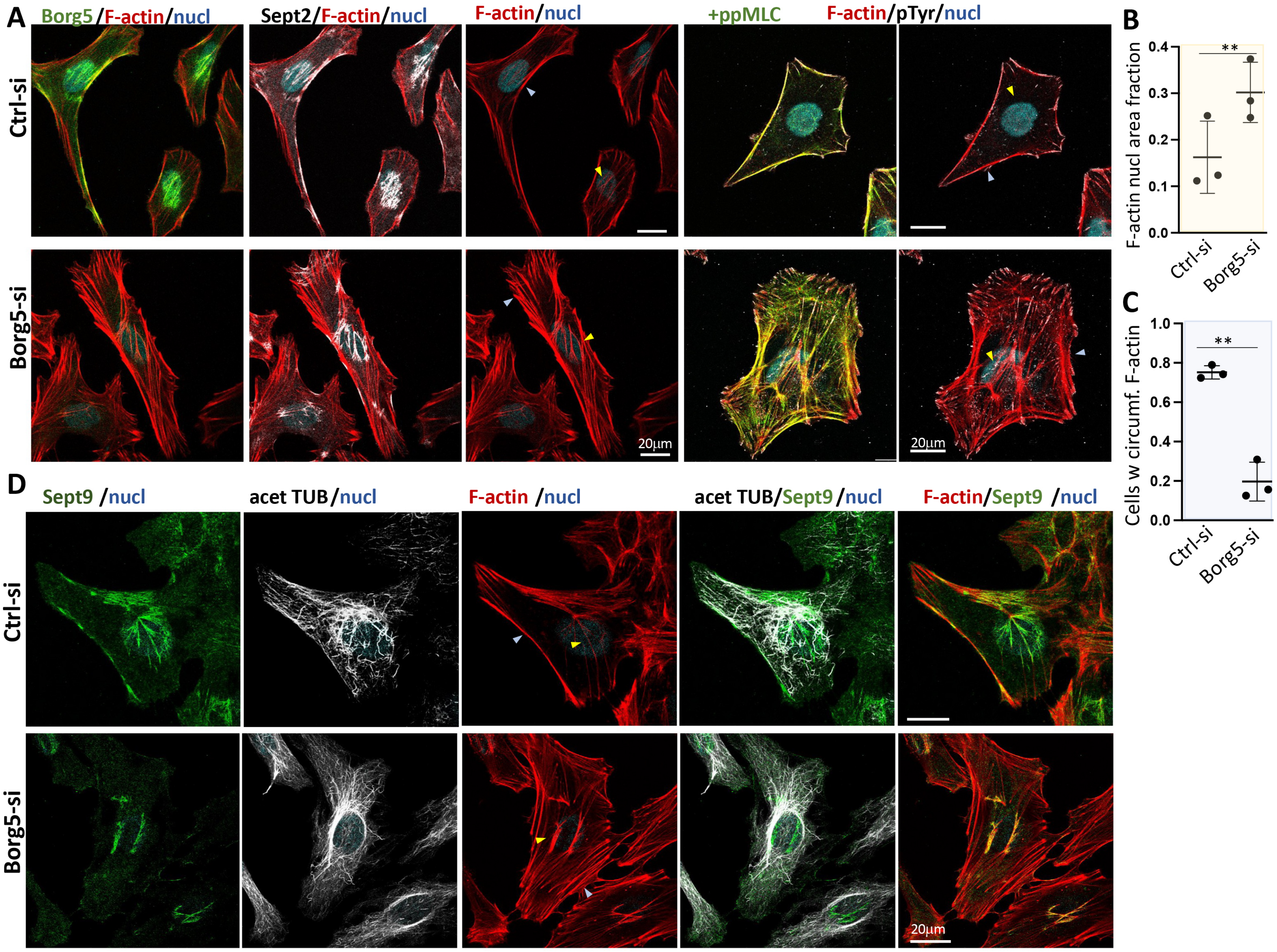

Fig. S6. Borg5 depletion in HeLa cells increases F-actin under the nucleus and prevents compressed cortical F-actin bundles

Control (Ctrl-si) or Borg5-depleted (Borg5-si) HeLa cells, cultured overnight at a density of 20,000 cells/cm^2 on collagen-coated coverslips were analyzed for the indicated markers. phospho-tyrosine (pTyr) labeling is concentrated in FAs. **A,D**) Yellow arrowheads indicate higher F-actin levels under the nucleus in Borg5-si compared to ctrl-si cells; blue arrowheads point to compact peripheral cortical F-actin in ctrl-RNAi treated cells versus uncompacted F-actin bundles in Borg5-RNAi treated cells. Note in A) that septin 2 (Sept2) accumulates under the nucleus independently of Borg5 and in D) that septin 9 (Sept9) maintains F-actin alignment and remains distinct from acetylated tubulin (acet TUB) in both ctrl conditions and upon Borg5-RNAi. **B**) F-actin nuclear area fraction was determined by overlaying a mask of the nucleus onto the F-actin channel of merged confocal planes below the nucleus; **C**) cell fraction with circumferential F-actin bundles; data in B,C) are derived from 8 random images with 5-8 cells each, N=3; B) **p=0.06; C) **p=0.005 by paired two-tailed *t*-test.

Fig.1F

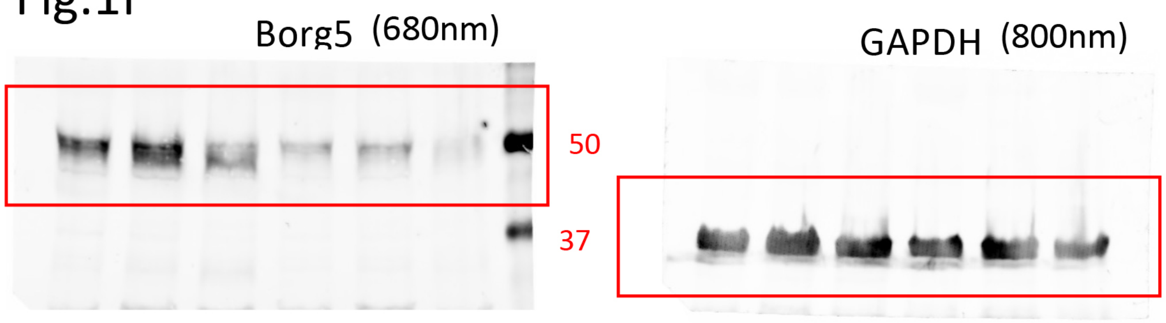

Fig.2A

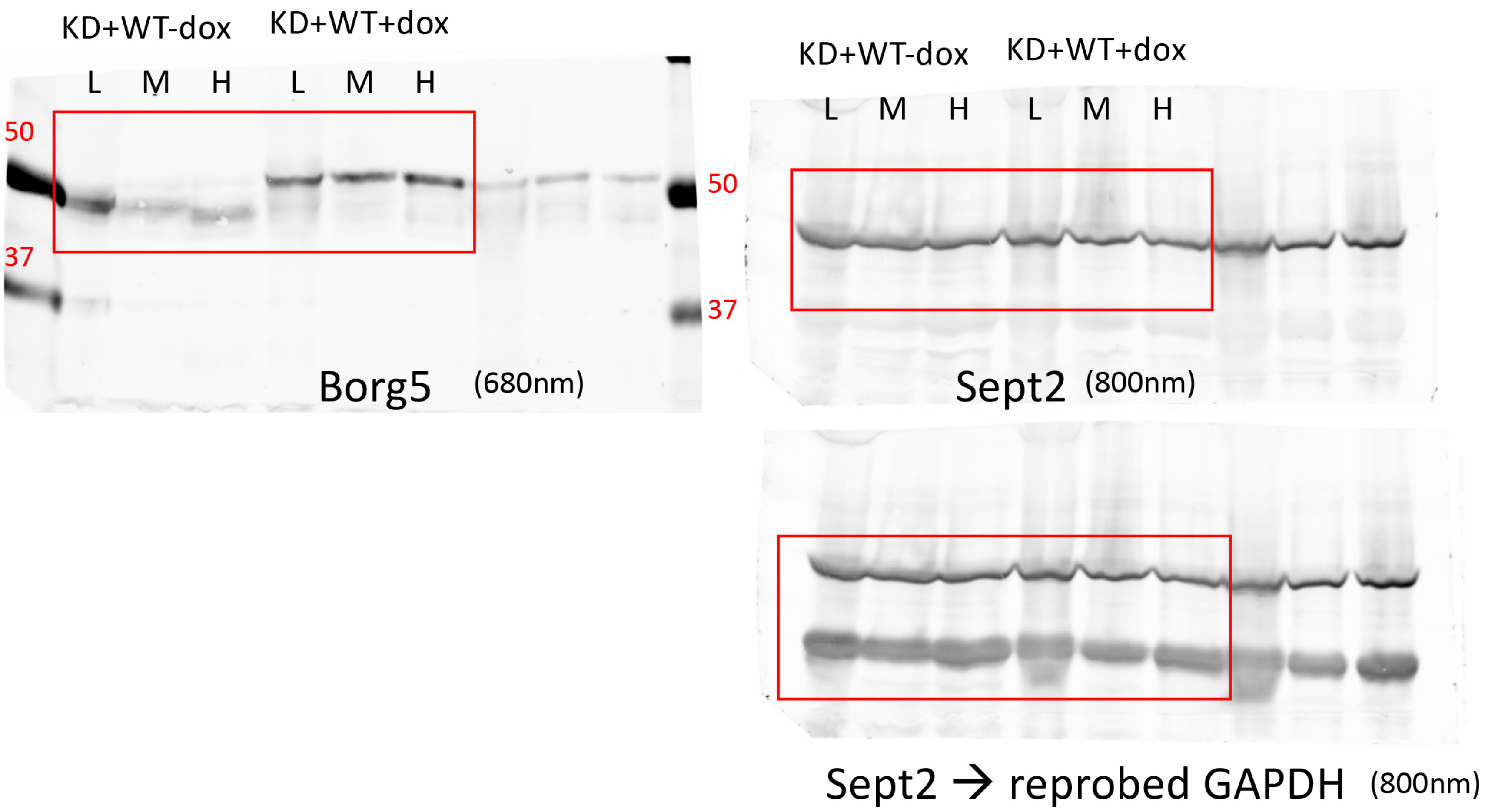

Fig. S5C

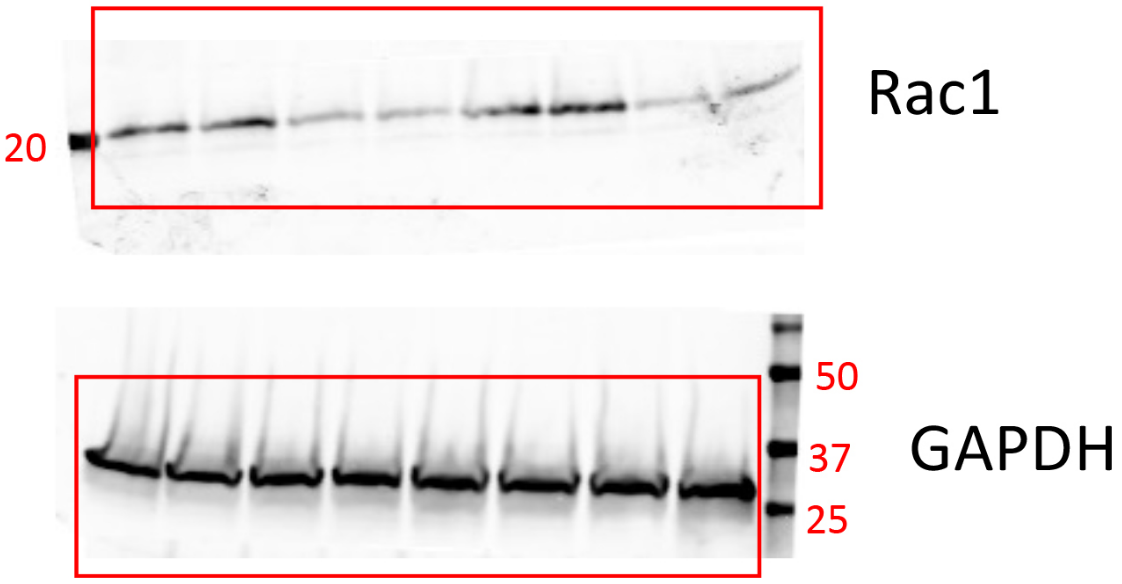

Journal of Cell Science • Supplementary information

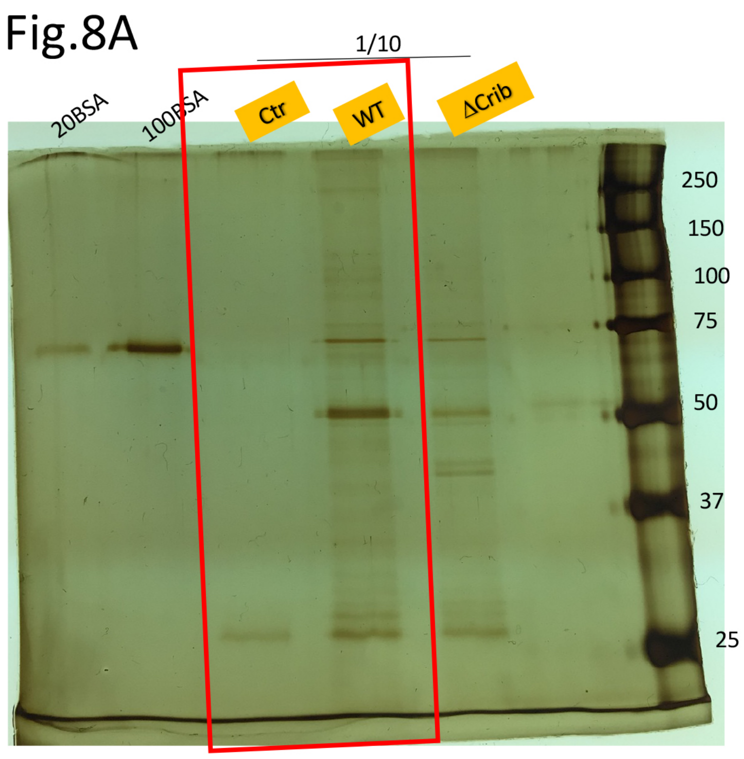

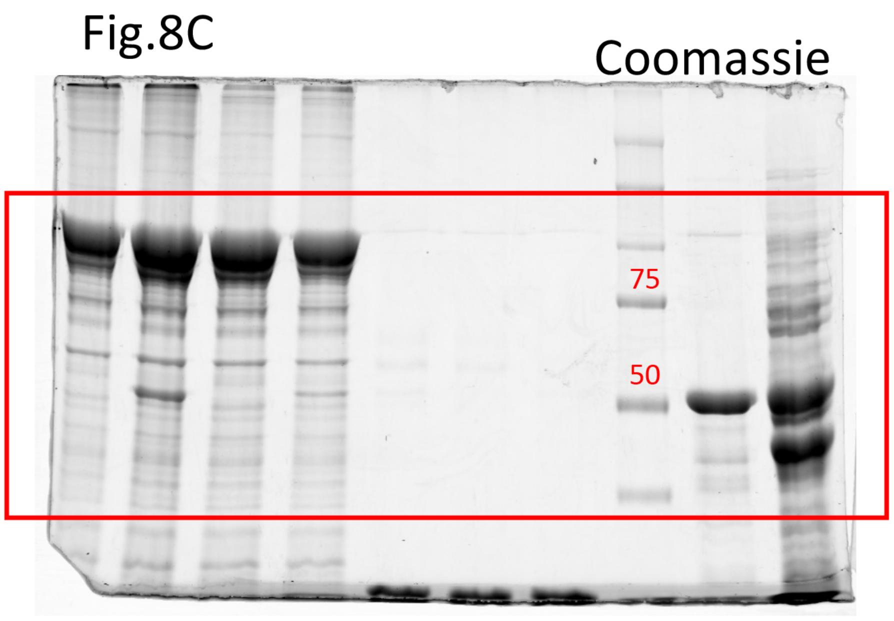

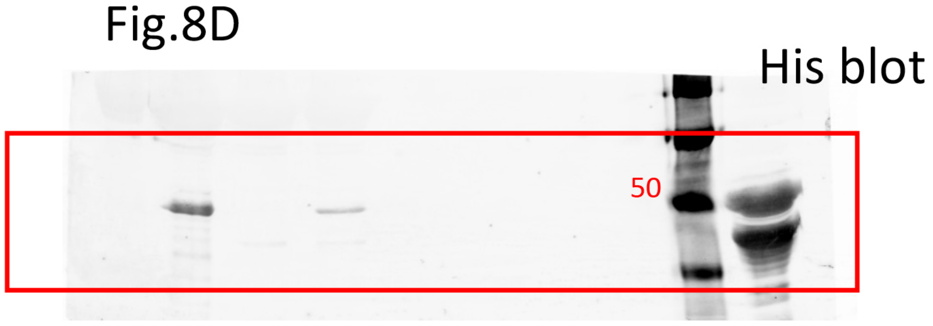

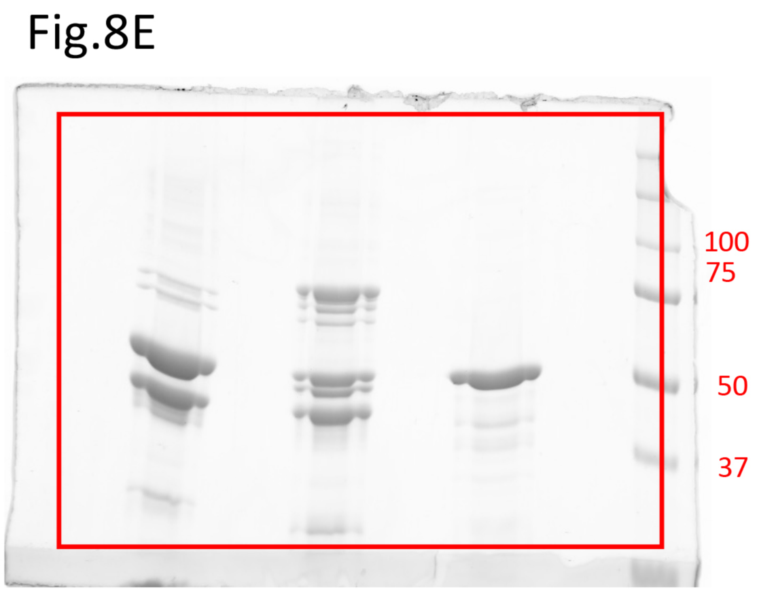

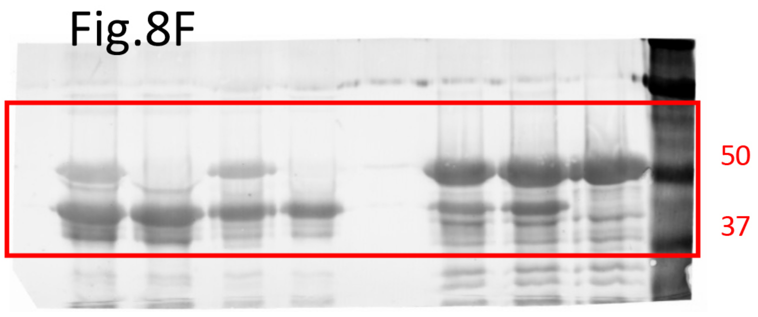

Journal of Cell Science • Supplementary information

Fig.8G

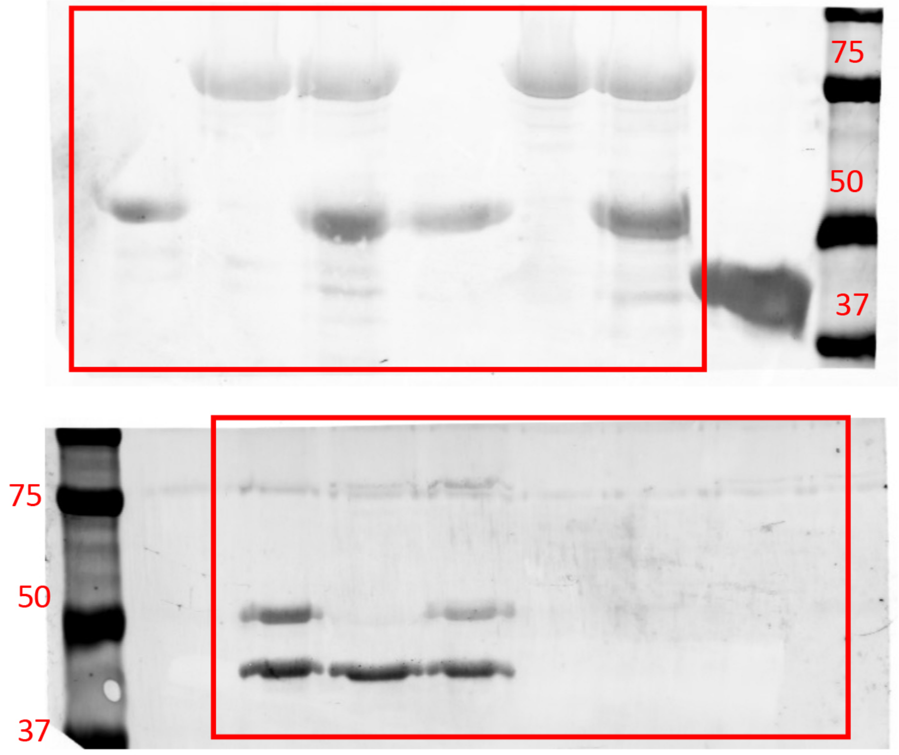

Fig.8H

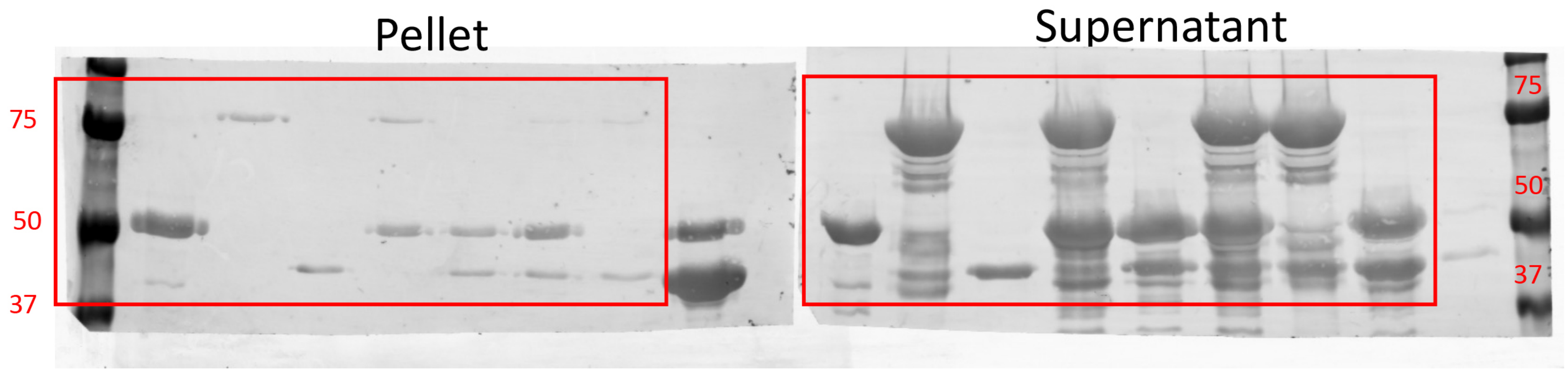

Fig. S7. Uncropped images of Coomassie Gels and Immunoblots

Red boxes frame part of images as presented in the figures and numbers in red refer to the size of molecular weight markers if apparent in the blot/gel.

Table S1. List of proteins co-isolated with Borg5-HALO from MDCK lysates

Sequence coverage and Average #PSM from 3 experiments for non-transfected control (C) and Borg5-HALO (W) transfected cell samples.

Available for download at
https://journals.biologists.com/jcs/article-lookup/doi/10.1242/jcs.261705#supplementary-data

Journal of Cell Science • Supplementary information

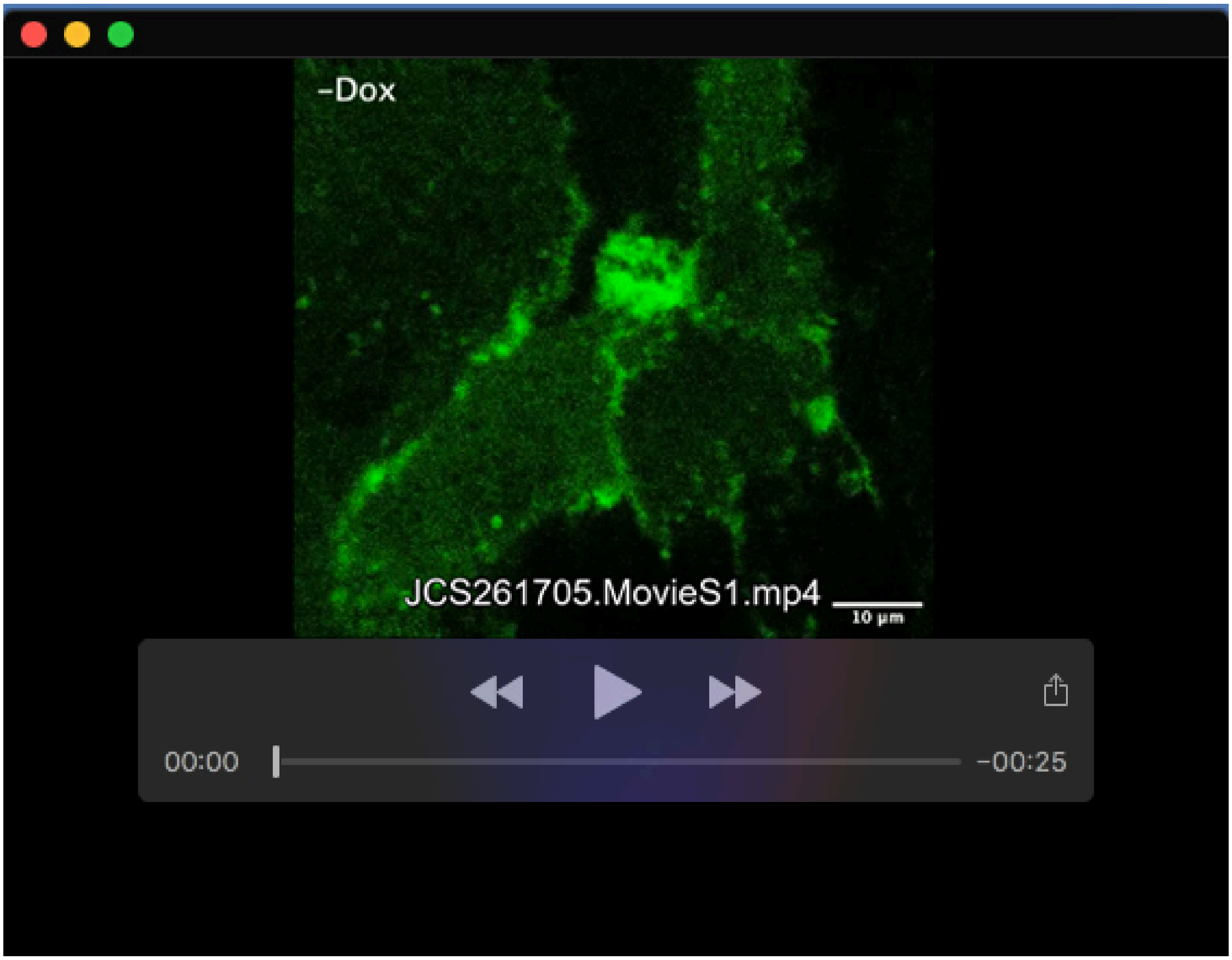

Movie 1. ACTN4 dynamics in Borg5KD cells. *(related to Fig.4).* Confocal *x-y-t* time-lapse (1frame/30sec, 6min -Dox, 10min +Dox) at the basal domain of ACTN4-GFP transfected Borg5-KD control (-Dox) and Borg5-KD (+Dox) cells in confluent monolayers, plated at $3x10^4$ cells/cm^2 and grown for 24 h. Bar=10µm

Journal of Cell Science • Supplementary information

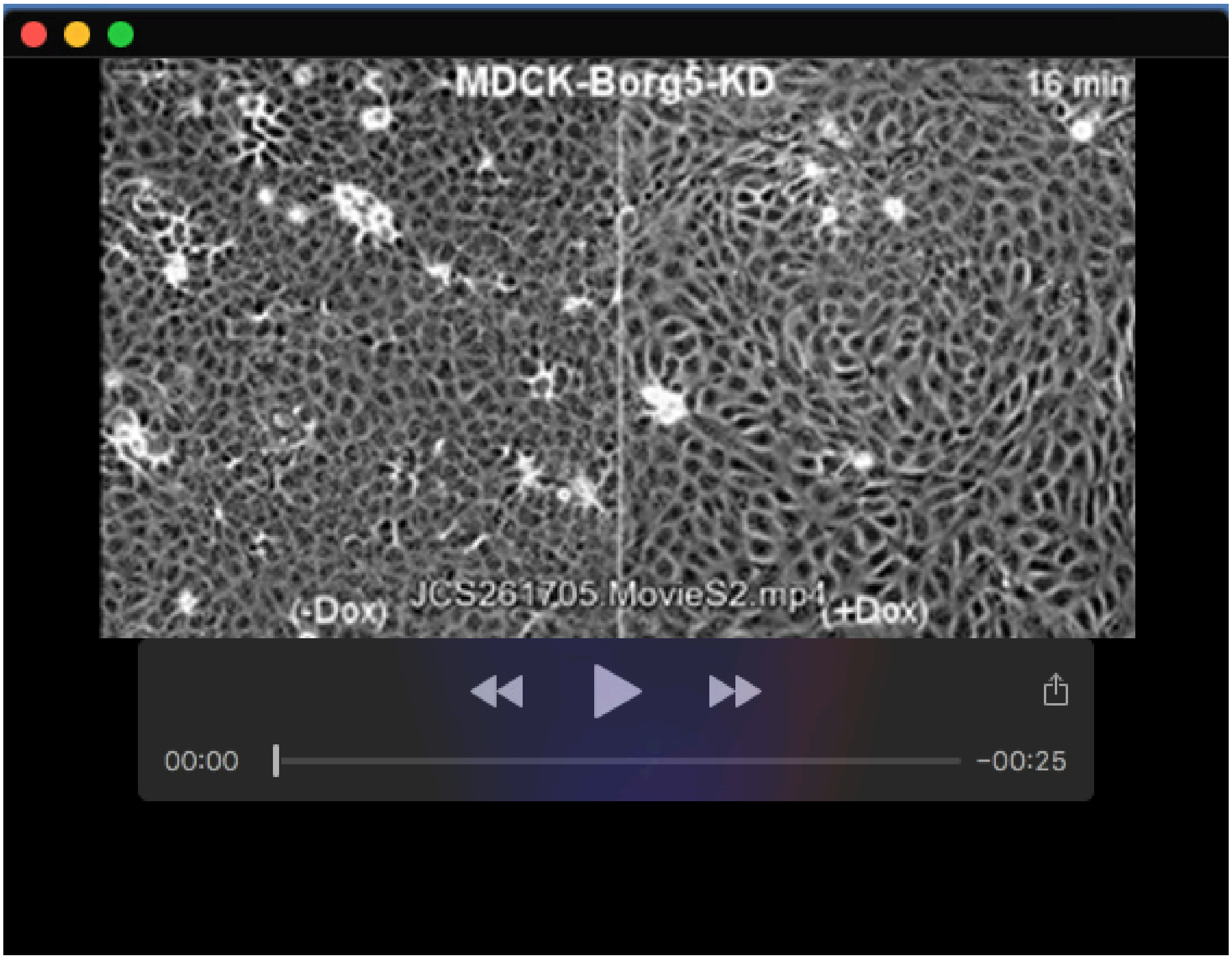

Movie 2. Streaming of Borg5-KD cells. (related to Fig. 5E). Phase contrast time lapse (1frame/5 min, 60min) of Borg5-KD control (-Dox) and Borg5-KD (+Dox) monolayers that were plated at $3x10^4$ cells/cm^2 and grown for 24 h. Bar=100 µm.

Journal of Cell Science • Supplementary information

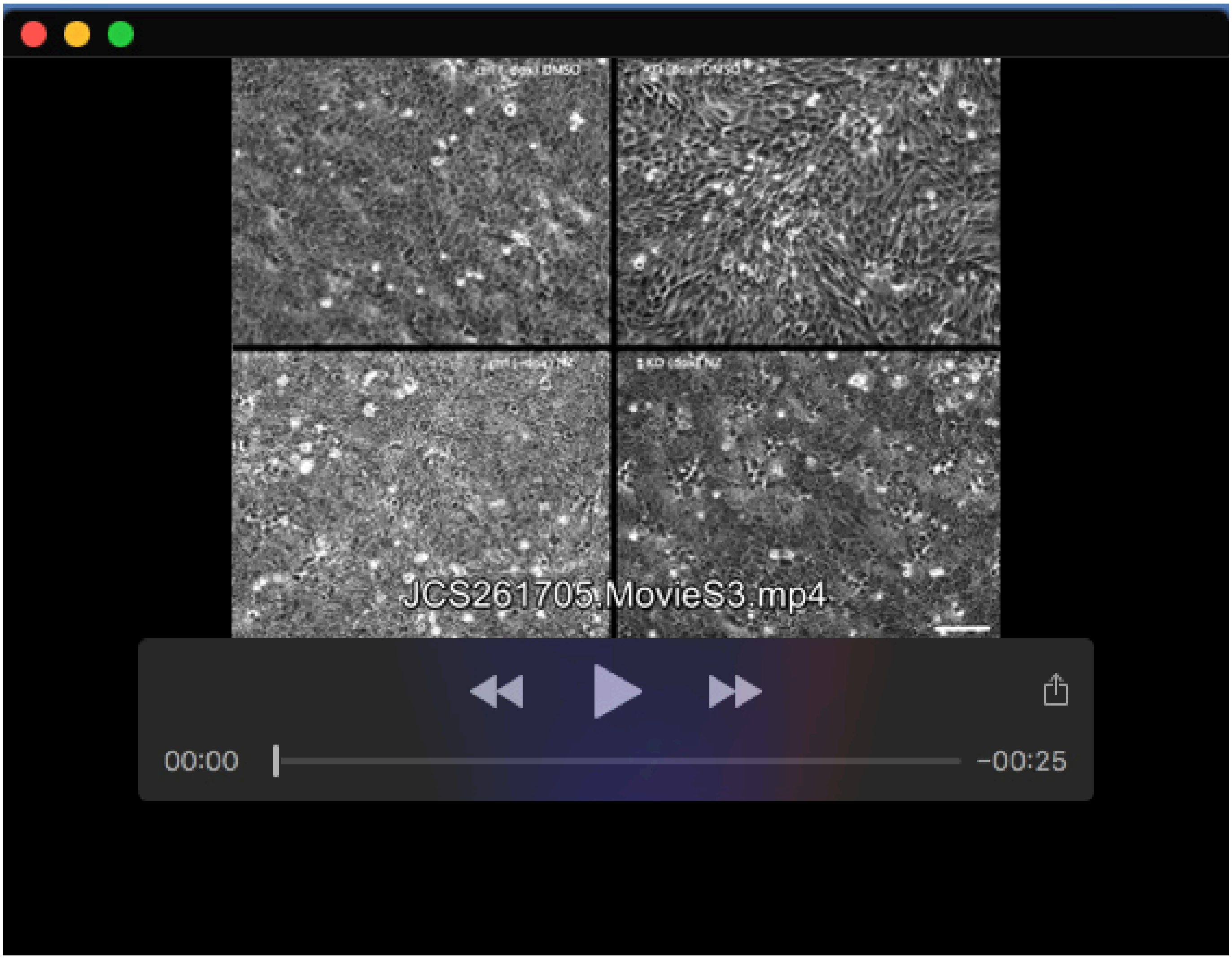

Movie 3. Effect of MT-disruption on Streaming of Borg5-KD cells. (related to Fig. 6A). Phase contrast time lapse (1frame/5min, 60min) of Borg5-KD control (-Dox) and Borg5-KD (+Dox) monolayers that were plated at $3x10^4$ cells/cm^2, grown for 24 h and treated with 10µM. Nocodazole or DMSO solvent for 2h prior imaging. Bar=100 µm.
