# Supplementary material for: Borg5 restricts contractility and motility in epithelial MDCK cells
Source: J Cell Sci. 2024 Dec 10;137(23):jcs261705. doi: 10.1242/jcs.261705 (PMC11698036; doi:10.1242/jcs.261705)
Supplement: Supplementary file 1 [file joces-137-261705_review_history.pdf]

Borg5 restricts contractility and motility in epithelial MDCK cells

David Cohen, Dawn Fernandez, Francisco Lázaro-Diéguez, Beatrix Überheide and Anne Müsch
DOI: 10.1242/jcs.261705

Editor: Kathleen Green

Review timeline
Original submission: 4 October 2023
Editorial decision: 7 November 2023
First revision received: 20 September 2024
Editorial decision: 14 October 2024
Second revision received: 22 October 2024
Accepted: 30 October 2024

Original submission

First decision letter

MS ID#: JOCES/2023/261705

MS TITLE: Borg5/Cdc42EP1 curbs Septin dependent stress fiber formation by antagonizing α-Actinin 4 and Myosin IIA

AUTHORS: David Cohen, Dawn Michelle Fernandez, Francisco Lazaro-Dieguez, Beatrix Ueberheide, and Anne Muesch

We have now reached a decision on the above manuscript.

To see the reviewers' reports and a copy of this decision letter, please go to: https://submit-jcs.biologists.org and click on the 'Manuscripts with Decisions' queue in the Author Area. (Corresponding author only has access to reviews.)

As you will see, the reviewers raise a number of substantial criticisms that prevent me from accepting the paper at this stage. They suggest, however, that a revised version might prove acceptable, if you can address their concerns. If you think that you can deal satisfactorily with the criticisms on revision, I would be pleased to see a revised manuscript. We would then return it to the reviewers.

Please ensure that you clearly highlight all changes made in the revised manuscript. Please avoid using 'Tracked changes' in Word files as these are lost in PDF conversion.

I should be grateful if you would also provide a point-by-point response detailing how you have dealt with the points raised by the reviewers in the 'Response to Reviewers' box. Please attend to all of the reviewers' comments. If you do not agree with any of their criticisms or suggestions please explain clearly why this is so.

Reviewer 1

© 2024. Published by The Company of Biologists under the terms of the Creative Commons Attribution License (https://creativecommons.org/licenses/by/4.0/).

Advance summary and potential significance to field

In this work, the authors describe a novel role for the septin-binding Borg5/Cdc42ep1 as a negative regulator of stress fibre assembly in MDCK cells. This is in contrast to several previous studies on Borgs which have mostly described them as positive regulators of actomyosin filaments. The authors begin by characterising the phenotypes associated with Borg5 depletion which include a dramatic increase in myosin activation and increased junctional tension. Using mass-spectrometry, the authors identify, then validate using biochemistry approaches, two novel binding partners of Borg5: myosin IIA and alpha-actinin 4. These are already established binding partners of septins. They then demonstrate that both septin 2 and septin 9 are required for the formation of stress fibers associated with Borg5 KD. The authors finally hypothesize that the ability of Borg5 to inhibit stress fibre formation is due to local competition with myosin IIA and ACTN4 for septin binding. This is an important piece of work as the role of Borg proteins in cytoskeletal dynamics remains largely uncharacterised.

Comments for the author

1. In Figure 1, the authors describe an increase in stress fibres, ppMLC and vinculin positive focal adhesions upon Borg5 knockdown in confluent MDCK cells. Vinculin however has also been reported to be associated with cell-cell junctions in MDCK cells in a myosin dependent manner (le Duc et al. 2010). In this regard, better visualization of vinculin along mid-plane junctions in Figure 1A would be ideal.

2. In Figure 2B, in addition to fluorescence intensity, measurements of changes in junctional width should also be performed. Examples displayed are strikingly different from Figure 1B where Borg5 KD resulted in very thick appearing E-cadherin positive junctions, and Figure 1A where Borg5 KD appears to lead to a strong reduction of F-Actin along junctions. These discrepancies should be addressed.

3. In Figure 4, images displaying cells transfected with the full length or CEP mutant of Borg5 do not appear strikingly different from their un-transfected neighbors. Visualization of myosin and/or junctional proteins would also be beneficial.

4. In Figure 7, the authors claim Sept2 and Sept9 are mediating the increase in ppMLC associated with Borg5 KD. While they describe changes in septin localization upon Borg5 KD, they do not comment about changes in septin expression. This may account for some of the observed phenotypes as septins have been previously reported to act as scaffolds for myosin activation (Joo et al. 2007).

5. The localization of Borg5 is mostly along a subset of actin filaments (Figure 3) and the images displaying negative correlation of Borg5 and stress fibres (Figure 8B) are not very convincing. How do the authors hypothesize that such a strong activation of myosin upon Borg5 KD as in Figure 8A, is based on local competition alone? The dramatic increase in stress fibres and myosin could be a result of highly activated RhoA. Indeed, early studies on overexpression of Borgs have suggested that they may play a role in inhibiting the function of RhoA (Joberty et al. 1999).

Overall this is an interesting study that demonstrates a novel function of Borg5, and with strong biochemistry identify previously uncharacterized direct binding partners. It should be noted though that the phenotypes observed may be cell-line specific, as other teams have on the contrary reported a reduction of ppMLC in endothelial cells upon knockout of Borg5 (Liu et al. 2014). In addition, given that knockdown of other Borgs has not been performed in MDCK cells, it cannot be assumed that this function is specific to Borg5 alone.

Reviewer 2

Advance summary and potential significance to field

In this manuscript, Cohen et al report a novel role for the CD42 effector protein 1 (Borg5) in the maintenance of a subset of actin stress fibers in MDCK epithelia.

© 2024. Published by The Company of Biologists under the terms of the Creative Commons Attribution License (https://creativecommons.org/licenses/by/4.0/).

The authors show that Borg5 knock-down alters the organization of the actin network in polarized MDCK epithelia which become more mesenchymal like with actin stress fiber attached to focal adhesions, which correlates with cell elongation and streaming. The polarized epithelia also become shorter with reduced apical and lateral actin. In search of the mechanism that underlies this Borg5-dependent maintenance of ventral actin network, the authors first show that Borg5 colocalizes with septins on ventral actin stress fiber under the cell nucleus.
Using proteomics analysis, they identify myosin IIA and a-actinin-4 (ACTN-4) as binding partners of Borg5 along with septins, which were lower abundance interactors. In competitive binding assays with purified recombinant proteins and cell extracts, the authors show that septins compete with Borg5 for binding to myosin and Borg5 competes with ACTN-4 for actin-binding. Interestingly, Borg5 depletion resulted in loss of septins from actin stress fibers, but septins were required for the formation of the alternate network of "stellate" stress fibers which was also dependent on myosin II, and ACTN-4, but not VASP and cofilin. These data are supported by microscopy results that show mutually exclusive localizations of septins/Borg5 with myosin/ACTN-4 and enrichment of myosin II an ACTN4 in the reorganized "stellate" stress fiber network of Borg4-depleted cells.

The authors have conducted a lot of work. Experiments and analyses are mostly well done. Though some of the imaging can be better with more zoomed-in, bigger and higher resolution images as it's hard to see some of the nuances in the small panels.

Conceptually, it remains unclear how Borg5 and septins synergize for the formation of a presumably non-contractile ventral actin stress fiber network underneath the nucleus. The physiological role of this network also remains unknown in the context of epithelial morphogenesis, and confounded by the reduction of apico-lateral actin network in polarized epithelia given that the latter is not investigated with respect to Borg5/septins.

Comments for the author

1) The entire work is conducted in MDCK cells, but a network of septin-positive ventral stress fibers that localize underneath the nucleus has been reported in a variety of other cell types. Is the main phenotype reproducible in other cell lines? Validation of major findings (actin network reorganization vis as vis septin/myosin/ACT4 localizations) in another cell type would be useful in enhancing the significance of the work, and ruling out the possibility of that these findings are cell type specific.

2) In attempt to increase the physiological relevance of the Borg5-dependent basal network – re: possible suppression of highly contractile migratory actin network – the authors should look at levels of endogenous Borg5 expression in MDCK cells at single cell density and/or partial EMT state (HGF-treated) versus polarizing and fully polarized states (time-course of polarization on transwells).
Is it possible that Borg5 expression increases during polarization, which may shift basal actin networks to a non-contractile morphology and organization?

3) The manuscript can improve with a better imaging of Borg5 localization to the different actin stress fiber networks with respect to septins, myosin IIA and ACTN-4. The images in Figure 3 are in sub-confluent monolayers, which is in contradiction with images in Figures 1-2 which are in more confluent monolayers.
Quantification of septin colocalization with Borg5 on basal actins stress fibers vs septin colocalization with myosin IIA, and ACTN-4 should be conducted to demonstrate that there is more Borg5 and septins on the basal actin network, than there is septins with myosin II or ACTN4. There part of the manuscript can be strengthened with additional high-/super-resolution imaging and quantification.
Have the authors looked at myosin IIB and IIC? Is is possible that septins colocalize with myosin IIB or IIC on the basal actin network? The lack of myosin IIA on septin-actin networks is surprising.

What happens if the full length Borg5 is over-expressed? Is there more work basal network without myosin/ACTN-4 formed? Do cells polarize faster?

4) The data on acetylated microtubules and the formation of neurite-like and other microtubule-based tubular structures are a major diversion from the main narrative of the paper.

© 2024. Published by The Company of Biologists under the terms of the Creative Commons Attribution License (https://creativecommons.org/licenses/by/4.0/).

The manuscript goes on a tangent, and the data do not amount to any new insight into the main focus of the paper. It is also unclear if some of these structures are intercellular bridges from failed abscission. The authors should remove this part of the manuscript. It convolutes the manuscript's main story, which is in itself fairly complex to grasp.

5) In Figure 5G, there is a lane 11 labeling, but there is nothing in that lane in the gel above. And in the western image of the gel, there is no 9 and 10 lane, but only 11. This is confusing. Why aren't all lanes shown in either gel images?

The biochemistry data are subject to the caveat of differential results at high and low salt conditions, which makes things more confusing for the reader. In addition, the weak interaction between septins and Borg5 further muddles the interpretation of the results.

Can the authors clarify whether septin-actin binding is enhanced in the presence of Borg5, reduced or whether Borg5 has no effect on septin-actin binding? On the other hand, does septin affect the actin-binding of Borg5? This important in clarifying the role of septins and Borg 5 in the formation of the basal non-stellate actin network. Especially since there is some unpublished work suggesting that septins scaffold actin formation via recruitment of actin monomers by Borgs.

6) In Figure 7, it is shown that Borg5 reduces septin colocalization with actin stress fibers. It is stated that in control cells most septin filaments are flanked by vinculin-positive focal contacts, which was reduced in Borg5 knock-down. This finding contradicts the findings of Figure 1, where Borg5 depletion enhances the stress fibers which are connected to vinculin focal adhesions, and the majority of ventral actin stress fibers (which presumably colocalize with septins) in control cells are not connected to vinculin positive focal adhesions.
It is hard to reconcile these two findings with one another as septin filaments that end to vinculin colocalize with actin stress fibers.

7) A graphical model/cartoon would be useful in demonstrating the authors conclusion/interpretation of collective data. This can be provided as a panel in last main figure or in supplementary figure.

Minor:
There are reference formatting issues throughout the document with references appearing in braces rather than parenthesis. These should be corrected.

First revision

<u>Author response to reviewers' comments</u>

Dear Kathleen,

We are re-submitting our revised Manuscript #JOCES/2023/261705. We are grateful to you for handling the submission and to both reviewers for their engaged and helpful critique, which we address point by point below (replies in red).

While working on the suggested revisions, a new commercial Borg5 antibody became available, which allowed us to detect endogenous MDCK Borg5 by Immunofluorescence (IF) for the first time. IF analysis of native Borg5 compared to that of the recombinantly expressed Borg5 we previously studied, brought several new insights now presented in the revised version.

The new findings are: (1) that Borg5 localization and expression levels change during MDCK polarization (now Fig.1); (2) that when overexpressed, Borg5 does not increase ventral stress fibers, despite recruiting Septins to these sites (now Fig. 2); (3) that both Borg5 overexpression and depletion alter apical cell shape independent of effects on stress fibers (now Fig. 7); (4) that Borg5 depletion decrease septin alignment with F-actin and causes microtubule stabilization (now Fig.s S2

© 2024. Published by The Company of Biologists under the terms of the Creative Commons Attribution License (https://creativecommons.org/licenses/by/4.0/).

and Fig. 4D) and (5) that streaming behavior and ruffles in Borg5-depleted cells are microtubule-dependent (now Fig.6).

We have removed data that the reviewers critiqued as unconvincing, specifically the Figure suggesting an inverse correlation between Borg5/septins and Myosin IIA; we also left out the expression of the Borg5 domain constructs, which we judged to contribute little to the mechanistic characterization of Borg5, and we eliminated the data related to the Borg5-ACTN4 interaction to focus solely on Myosin II for which we have stronger interaction data and more convincing connections to the phenotype.

Additional changes are explained in the response to individual critique points:

Reviewer 1 Comments for the Author:
1.In Figure 1, the authors describe an increase in stress fibres, ppMLC and vinculin positive focal adhesions upon Borg5 knockdown in confluent MDCK cells. Vinculin however has also been reported to be associated with cell-cell junctions in MDCK cells in a myosin dependent manner (le Duc et al. 2010). In this regard, better visualization of vinculin along mid-plane junctions in Figure 1A would be ideal.

We have tested Vinculin labeling in confluent monolayers using two different commercial Vinculin antibodies. However, in our hands too little Vinculin is apparent in midplanes in Borg5-KD cells to establish a firm difference to control cells and thus, we have not included it.

2.In Figure 2B, in addition to fluorescence intensity, measurements of changes in junctional width should also be performed. Examples displayed are strikingly different from Figure 1B where Borg5 KD resulted in very thick appearing E-cadherin positive junctions, and Figure 1A where Borg5 KD appears to lead to a strong reduction of F-Actin along junctions. These discrepancies should be addressed.

We have removed junctional intensity measurements from the revised version.

3.In Figure 4, images displaying cells transfected with the full length or CEP mutant of Borg5 do not appear strikingly different from their un-transfected neighbors. Visualization of myosin and/or junctional proteins would also be beneficial.

We removed the Borg5 mutant analysis from the revised version to make room for the new analysis of endogenous Borg5. We decided it yielded limited insight as we have not linked specific phenotypic effects to individual Borg5 domains.

4.In Figure 7, the authors claim Sept2 and Sept9 are mediating the increase in ppMLC associated with Borg5 KD. While they describe changes in septin localization upon Borg5 KD, they do not comment about changes in septin expression. This may account for some of the observed phenotypes as septins have been previously reported to act as scaffolds for myosin activation (Joo et al. 2007).

We have now tested this idea and included data in Fig.2A, showing that although Borg5 levels, particularly Borg5 overexpression, alter Septin 2 distribution (Fig.2B-D), they do not alter total Septin 2 levels.

5.The localization of Borg5 is mostly along a subset of actin filaments (Figure 3) and the images displaying negative correlation of Borg5 and stress fibres (Figure 8B) are not very convincing. How do the authors hypothesize that such a strong activation of myosin upon Borg5 KD as in Figure 8A, is based on local competition alone? The dramatic increase in stress fibres and myosin could be a result of highly activated RhoA. Indeed, early studies on overexpression of Borgs have suggested that they may play a role in inhibiting the function of RhoA (Joberty et al. 1999).

We agree with the reviewer that the stark increase in contractility in Borg5 depleted cells might have additional or even different causes than the Borg5 competition with septin-myoII binding/activation we suggested. We are now discussing the competition as one possible mechanism

© 2024. Published by The Company of Biologists under the terms of the Creative Commons Attribution License (https://creativecommons.org/licenses/by/4.0/).

rather than a likely mechanism for the stress fiber phenotype. We have removed Figure 8B and left Borg5's role in Myosin regulation out of the title of manuscript.
The increased MyoII activity is indeed Rho/RhoK-dependent as the reviewer suggested, which is shown in current Fig. S3.

Overall this is an interesting study that demonstrates a novel function of Borg5, and with strong biochemistry, identify previously uncharacterized direct binding partners. It should be noted though that the phenotypes observed may be cell-line specific, as other teams have on the contrary reported a reduction of ppMLC in endothelial cells upon knockout of Borg5 (Liu et al. 2014). In addition, given that knockdown of other Borgs has not been performed in MDCK cells, it cannot be assumed that this function is specific to Borg5 alone.

We agree with this statement. Regarding other Borg family members in MDCK cells, we were unable to detect expression of Borg4, which along with Borg5 was reported in MDCK proteomics analysis; we could thus not address the role of other putative MDCK Borg proteins. However, we have now analyzed the effect of Borg5 depletion in HeLa cells, which express high levels of Borg5. We detected stress fiber abnormalities comparable to those in Borg5-depleted MDCK cells. These finding are now presented in Fig. S6. We have now also included a discussion of possible reasons for apparent cell type specific differences in Borg5 depletion in the DISCUSSION section.
*****

Reviewer 2 Comments for the Author:
1)The entire work is conducted in MDCK cells, but a network of septin-positive ventral stress fibers that localize underneath the nucleus has been reported in a variety of other cell types. Is the main phenotype reproducible in other cell lines? Validation of major findings (actin network reorganization vis as vis septin/myosin/ACT4 localizations) in another cell type would be useful in enhancing the significance of the work and ruling out the possibility of that these findings are cell type specific.

We have addressed this question by depleting Borg5 from HeLa cells, which expressed high levels of Borg5 and have ventral stress fibers. We found that they likewise increase sub-nuclear stress fibers upon Borg5 depletion and exhibit less compacted cortical F-actin. These findings are now presented in Fig. S6. Nevertheless, the reported decrease in MyoII activity in vascular endothelial cells in Borg5 KO mice (Liu et al. 2014), suggest that there are indeed cell-type specific differences to the role of Borg5. We now discuss this in the DISCUSSION section of the manuscript.

2)In attempt to increase the physiological relevance of the Borg5-dependent basal network – re: possible suppression of highly contractile migratory actin network – the authors should look at levels of endogenous Borg5 expression in MDCK cells at single cell density and/or partial EMT state (HGF-treated) versus polarizing and fully polarized states (time-course of polarization on transwells).Is it possible that Borg5 expression increases during polarization, which may shift basal actin networks to a non-contractile morphology and organization?

We have only recently obtained a Borg5 antibody suitable for the robust detection of Borg5 in MDCK cells and analyzed Borg5 levels and distribution at different stages of polarization, including on transwells (Fig.1). We found that Borg5 levels decrease with polarization and shift from stress fiber -associated and (lateral) cortical population in single and polarizing contacting cells to a Zonula adherens -associated and an apical population in fully polarized cells. (Fig.1). Thus, stress fiber localization ceases as cells experience fewer traction forces. Because of its predominant apical localization in polarized cells, we have now also included our data describing a role for Borg5 in the regulation of apical surface morphology in mature monolayers (Fig.7).

3)The manuscript can improve with a better imaging of Borg5 localization to the different actin stress fiber networks with respect to septins, myosin IIA and ACTN-4. The images in Figure 3 are in sub-confluent monolayers, which is in contradiction with images in Figures 1-2 which are in more confluent monolayers.
Quantification of septin colocalization with Borg5 on basal actins stress fibers vs septin colocalization with myosin IIA, and ACTN-4 should be conducted to demonstrate that there is more Borg5 and septins on the basal actin network, than there is septins with myosin II or ACTN4. There

© 2024. Published by The Company of Biologists under the terms of the Creative Commons Attribution License (https://creativecommons.org/licenses/by/4.0/).

part of the manuscript can be strengthened with additional high-/super-resolution imaging and quantification.
Have the authors looked at myosin IIB and IIC? Is is possible that septins colocalize with myosin IIB or IIC on the basal actin network? The lack of myosin IIA on septin-actin networks is surprising.

We have included better and larger images describing the endogenous localization of Borg5, particularly with regard to the actin cytoskeleton and septins. Regarding the colocalization analysis: We had previously presented colocalization analysis with recombinantly expressed proteins demonstrating that septins and Borg5 colocalized better with each other than either one with MyosinII or ACTN4. However, even for septin and Borg5 the Manders and Pearsons coefficients were low. Recent super-resolution imaging of F-actin and septins by Mavrakis' lab (Martins et al., 2023) showed that septin and actin filaments are not precisely colocalized but rather entwined. We have thus abstained from quantitative pixel-by-pixel colocalization analysis of septins with F-actin and myosin II and, in the revised version do not claim that MyosinIIA is not localized to septin-actin networks.

What happens if the full length Borg5 is over-expressed? Is there more work basal network without myosin/ACTN-4 formed? Do cells polarize faster?

We now show that Borg5 overexpression does not alter stress fiber formation or columnarization of MDCK monolayers. However, high Borg levels induce apical stress fibers and distort apical surfaces (Fig.7).

4)The data on acetylated microtubules and the formation of neurite-like and other microtubule-based tubular structures are a major diversion from the main narrative of the paper. The manuscript goes on a tangent, and the data do not amount to any new insight into the main focus of the paper. It is also unclear if some of these structures are intercellular bridges from failed abscission. The authors should remove this part of the manuscript. It convolutes the manuscript's main story, which is in itself fairly complex to grasp.

We no longer emphasize the neurite-like extensions and intercellular bridges. However, further investigation into the role of MTs in the Borg5 phenotype revealed that the formation of ACTN4-positive ruffles, cell stretching and streaming behavior in Borg5-depleted monolayers are indeed MT-dependent (Figs.4F,G, Fig.6, video 4). Together with data that Borg5 depletion diminishes the alignment of septin with F-actin (Fig.S2) and increases stabilized MTs which partially colocalize with Septins (Fig.4D,E), we propose that one consequence of Borg5 depletion is to shift septin association from F-actin to MTs. The new work makes MTs a major contributor to the phenotypic hallmarks of Borg5-depletion.

5)In Figure 5G, there is a lane 11 labeling, but there is nothing in that lane in the gel above. And in the western image of the gel, there is no 9 and 10 lane, but only 11. This is confusing. Why aren't all lanes shown in either gel images?

We have fixed this.

The biochemistry data are subject to the caveat of differential results at high and low salt conditions, which makes things more confusing for the reader. In addition, the weak interaction between septins and Borg5 further muddles the interpretation of the results.

Can the authors clarify whether septin-actin binding is enhanced in the presence of Borg5, reduced or whether Borg5 has no effect on septin-actin binding? On the other hand, does septin affect the actin-binding of Borg5? This important in clarifying the role of septins and Borg 5 in the formation of the basal non-stellate actin network. Especially since there is some unpublished work suggesting that septins scaffold actin formation via recruitment of actin monomers by Borgs.

Unfortunately, we could not resolve if Borg5 binds actin. Borg5 IP did not yield any co-IP of actin and due to the tendency of Borg5 to aggregate, we were unable to establish interaction of Borg5 with F-actin in co-pelletation assays.

© 2024. Published by The Company of Biologists under the terms of the Creative Commons Attribution License (https://creativecommons.org/licenses/by/4.0/).

6)In Figure 7, it is shown that Borg5 reduces septin colocalization with actin stress fibers. It is stated that in control cells most septin filaments are flanked by vinculin-positive focal contacts, which was reduced in Borg5 knock-down. This finding contradicts the findings of Figure 1, where Borg5 depletion enhances the stress fibers which are connected to vinculin focal adhesions, and the majority of ventral actin stress fibers (which presumably colocalize with septins) in control cells are not connected to vinculin positive focal adhesions.
It is hard to reconcile these two findings with one another as septin filaments that end to vinculin colocalize with actin stress fibers.

We dropped the data in Fig.7 for new data, which we believe better characterize the Borg5-KD phenotype, but what was presented is indeed correct: Unlike the ventral stress fibers in controls, the Borg5-KD induced stress fibers are not aligned with septins (which is apparent in current Fig.4D) even though both stress fiber types are Vinculin anchored.

7)A graphical model/cartoon would be useful in demonstrating the authors conclusion/interpretation of collective data. This can be provided as a panel in last main figure or in supplementary figure.

We have not added a cartoon model but we have included descriptive models in the Discussion for the 3 separate Borg5 functions we describe based on Borg5's localization changes during the development of polarity.

Minor:
There are reference formatting issues throughout the document with references appearing in braces rather than parenthesis. These should be corrected.

We have fixed this.

We believe that these revisions have improved the manuscript and hope it is now suitable for publication in JCS.

Second decision letter

MS ID#: JOCES/2023/261705

MS TITLE: Borg5/Cdc42EP1 Restricts Contractility and Motility in Epithelial MDCK Cells

AUTHORS: David Cohen, Dawn Michelle Fernandez, Francisco Lazaro-Dieguez, Beatrix Ueberheide, and Anne Muesch

We have now reached a decision on the above manuscript.

To see the reviewers' reports and a copy of this decision letter, please go to: https://submit-jcs.biologists.org and click on the 'Manuscripts with Decisions' queue in the Author Area. (Corresponding author only has access to reviews.)

As you will see, the reviewers gave favourable reports but raised some critical points that will require amendments to your manuscript. I hope that you will be able to carry these out because I would like to be able to accept your paper, depending on further comments from reviewers.

Please ensure that you clearly highlight all changes made in the revised manuscript. Please avoid using 'Tracked changes' in Word files as these are lost in PDF conversion.

I should be grateful if you would also provide a point-by-point response detailing how you have dealt with the points raised by the reviewers in the 'Response to Reviewers' box. Please attend to all of the reviewers' comments. If you do not agree with any of their criticisms or suggestions please explain clearly why this is so.

© 2024. Published by The Company of Biologists under the terms of the Creative Commons Attribution License (https://creativecommons.org/licenses/by/4.0/).

Reviewer 1

Advance summary and potential significance to field

The authors have adequately addressed my comments and provided some interesting new insights. In my opinion it is acceptable for publication in Journal of Cell Science.

Comments for the author

The authors have adequately addressed my comments and provided some interesting new insights. In my opinion it is acceptable for publication in Journal of Cell Science.

Reviewer 2

Advance summary and potential significance to field

The authors have sufficiently addressed this reviewer's previous comments by adding/removing data.

Comments for the author

There are a few things that need be addressed in text and figures of the manuscript:

1. Not all references after a sentence are given in a single parenthesis with ; between individuals references. This needs to be addressed. Some references are shown individually in parenthesis followed by another parenthesis containing several references.

2. SEPT9_v1 should change to SEPT9_i1 wherever it is referred to as a protein. The v1 notation refers to mRNA transcripts. The correct notation for protein isoforms in the septin field is the use of i for isoform. V is for RNA transcript. Please correct throughout the entire text and figures.

3. The italicized notations bas and ap for basal and apical are confusing in figures. The italicized text made them read like a mutated gene. Please, consider writing basal and apical in plain text in all figure panels. It's much easier to look at the figures and understand what's going on. There are too many abbreviations as is with all the protein names etc.

4. There are parts of the Introduction which are not factually accurate with what's known in the field and what's published in total and need revision. They misrepresent previous findings:

a) "while Septin 2 competitively removes MAP4 from MTs (Kremer et al, 2005), which makes MT less stable and facilitates cargo transport (Spiliotis et al, 2008)"

Kremer et al argued that septins bind MAP4 and prevent it from binding microtubules, making microtubules more "dynamic"/less "stable". Subsequent work from multiple groups indicates that septins "stabilize" microtubules... they either promote microtubule growth by suppressing microtubule catastrophe/polymerization or by stunting microtubule growth. The terms "stable" and "dynamic" are problematic for microtubule field, because microtubules always have a degree of dynamicity.

Spiliotis et al, 2008 did not show that septins make microtubules less stable/more dynamic, just that facilitate Golgi to plasma membrane transport during polarization. Subsequent studies addressed septin functions in microtubule dynamics (see Bowen et al, and Nakos et al, papers). There are a lot of nuances and possibly septin subunit-dependent differences in the microtubule work especially in work that did not directly assay microtubule dynamics using in vitro TIRF motility assays or EB1 dynamics, and made extrapolations based on post-translational markers like acetylation. Please, revise to reflect what each paper showed and what the general view is in the field (for a more comprehensive review on this, see Spiliotis & Nakos review in Current Biology).

© 2024. Published by The Company of Biologists under the terms of the Creative Commons Attribution License (https://creativecommons.org/licenses/by/4.0/).

b) "Septin 9 competes with myosin for actin binding and recruits the actin-severing protein cofilin (Smith et al, 2015)"

The cited paper showed that Septin 9 competes with ATP-bound myosin (weakly actin-bound state) for actin binding. The cited paper showed that Septin 9 protects actin depolymerization by cofilin, and suggested that there is competition between cofilin and Sept9 for binding actin. There were NO data showing recruitment of cofilin by Septin 9. To the contrary, competition is likely to occur.

Second revision

Author response to reviewers' comments

We thank the reviewers for time and effort spent reviewing our revised manuscript. We have now addressed all points Reviewer 2 has raised. Below is our point-by-point response:

Reviewer 2 Comments for the Author:
There are a few things that need be addressed in text and figures of the manuscript:

1. Not all references after a sentence are given in a single parenthesis with ; between individuals references. This needs to be addressed. Some references are shown individually in parenthesis followed by another parenthesis containing several references.

We have fixed this and now present all references that belong together within a single bracket.

2. SEPT9_v1 should change to SEPT9_i1 wherever it is referred to as a protein. The v1 notation refers to mRNA transcripts. The correct notation for protein isoforms in the septin field is the use of i for isoform. V is for RNA transcript. Please, correct throughout the entire text and figures.

Thank you for making us aware of this convention; we have corrected this as well.

3. The italicized notations bas and ap for basal and apical are confusing in figures. The italicized text made them read like a mutated gene. Please, consider writing basal and apical in plain text in all figure panels. It's much easier to look at the figures and understand what's going on. There are too many abbreviations as is with all the protein names etc.

We have now replaced all italicized labeling “bas” and “Ap” with “basal” and “apical” in plain text

4. There are parts of the Introduction which are not factually accurate with what's known in the field and what's published in total and need revision. They misrepresent previous findings:

a) "while Septin 2 competitively removes MAP4 from MTs (Kremer et al, 2005), which makes MT less stable and facilitates cargo transport (Spiliotis et al, 2008)"

Kremer et al argued that septins bind MAP4 and prevent it from binding microtubules, making microtubules more "dynamic"/less "stable". Subsequent work from multiple groups indicates that septins "stabilize" microtubules... they either promote microtubule growth by suppressing microtubule catastrophe/polymerization or by stunting microtubule growth. The terms "stable" and "dynamic" are problematic for microtubule field, because microtubules always have a degree of dynamicity.

Spiliotis et al, 2008 did not show that septins make microtubules less stable/more dynamic, just that facilitate Golgi to plasma membrane transport during polarization. Subsequent studies addressed septin functions in microtubule dynamics (see Bowen et al, and Nakos et al, papers). There are a lot of nuances and possibly septin subunit-dependent differences in the microtubule work, especially in work that did not directly assay microtubule dynamics using in vitro TIRF

© 2024. Published by The Company of Biologists under the terms of the Creative Commons Attribution License (https://creativecommons.org/licenses/by/4.0/).

motility assays or EB1 dynamics, and made extrapolations based on post-translational markers like acetylation. Please, revise to reflect what each paper showed and what the general view is in the field (for a more comprehensive review on this, see Spiliotis & Nakos review in Current Biology).

The reviewer is right that the Spiliotis et al, 2008 citation did not report on the effect of Septin-MAP4 binding for MT-stability. We have therefore removed it from the Septin2/MAP4 discussion in this context.
We also now state the claim of the Kremer et al. study more specifically. As an introduction to Borg5, our intention is not to provide a comprehensive review of the evolution of the Septin-MT field; instead, by mentioning deacetylation and by contrasting the MAP4 data with those characterizing Septin 9_i1 as a MAP, we want to elude to the apparent complexities and multitudes of septins' scaffolding functions. We want to forward the idea that septin functions are likely subject to yet unkown regulatory mechanisms, which might involve Borg proteins.

b) "Septin 9 competes with myosin for actin binding and recruits the actin-severing protein cofilin (Smith et al, 2015)"

The cited paper showed that Septin 9 competes with ATP-bound myosin (weakly actin-bound state) for actin binding. The cited paper showed that Septin 9 protects actin depolymerization by cofilin,and suggested that there is competition between cofilin and Sept9 for binding actin. There were NO data showing recruitment of cofilin by Septin 9. To the contrary, competition is likely to occur.

Thank you for pointing out this mistake. We removed the reference to cofilin; the emphasis here was on the effect on myosin.

We hope the manuscriot is now acceptable for publication.

<u>Third decision letter</u>

MS ID#: JOCES/2023/261705

MS TITLE: Borg5/Cdc42EP1 Restricts Contractility and Motility in Epithelial MDCK Cells

AUTHORS: David Cohen, Dawn Michelle Fernandez, Francisco Lazaro-Dieguez, Beatrix Ueberheide, and Anne Muesch

I am happy to tell you that your manuscript has been accepted for publication in Journal of Cell Science, pending standard publication integrity checks.

© 2024. Published by The Company of Biologists under the terms of the Creative Commons Attribution License (https://creativecommons.org/licenses/by/4.0/).
